# The 3D-ASCr scale: A revalidation of the core dimensions of the Altered States of Consciousness Rating Scale 5D(11)-ASC for psychedelic research

**DOI:** 10.1177/02698811251397328

**Published:** 2025-12-26

**Authors:** Kurt Stocker, Matthias Hartmann, Yasmin Schmid, Severin B. Vogt, Anna M. Becker, Laura Ley, Isabelle Straumann, Denis Arikci, Aaron Klaiber, Livio Erne, Patrick Vizeli, Friederike Holze, Matthias E. Liechti

**Affiliations:** 1 Psychopharmacology Research, Division of Clinical Pharmacology & Toxicology, Department of Clinical Research, University Hospital Basel, University of Basel, Switzerland; 2 Chair of Cognitive Science, Department of Humanities, Social and Political Sciences, ETH Swiss Federal Institute of Technology Zurich, Switzerland; 3 Department of Psychology, University of Zurich, Switzerland; 4 Faculty of Psychology, UniDistance Suisse, Brig, Switzerland

**Keywords:** LSD, psilocybin, DMT, mescaline, psychedelics

## Abstract

**Background:**

The Altered States of Consciousness Scale (3/5D-ASC or 11-ASC) is widely used to assess non-ordinary states of consciousness, particularly for psychedelic research. However, its original dimensional model (3D-ASC within 5D-ASC) and later 11-subscale structure (11-ASC) have a hierarchically incompatible higher/lower-order structure. Although the 11-ASC offers superior model fit, the 3D-ASC remains widely used for summarizing broader experiential domains.

**Aims:**

We wanted to provide an updated, psychometrically revalidated version of the ASC. We tested whether the 42-item 11-ASC could be integrated into a coherent three-dimensional framework. We further hypothesized that this revised model would outperform the original 66-item 3D-ASC while preserving its conceptual clarity.

**Methods:**

Data from 901 5D-ASC questionnaires from 398 healthy participants across 16 randomized, mostly placebo-controlled psychedelic (lysergic acid diethylamide, psilocybin, mescaline, and *N*,*N*-dimethyltryptamine) studies were split for exploratory and confirmatory factor analysis. We compared the 3D-ASC and 11-ASC in terms of reliability and model fit, and tested whether the 11-ASC could be summarized within a three-dimensional model.

**Results:**

Ten of the 11 subscales formed three higher-order dimensions—Positive (PosE), Distressing (DisE), and Perceptual (PerE) effects—mirroring the 3D-ASC but with improved fit. We propose this as the 3D-ASCr scale. The Anxiety subscale could not be integrated due to consistent floor effects (low anxiety in the sample), but given its clinical relevance, it is retained within 3D-ASCr (as part of DisE or a standalone subscale).

**Conclusion:**

The 3D-ASCr is an updated version of the ASC and is recommended for use with classic serotonergic psychedelics in both clinical practice and research.

## Introduction

This article sets out to psychometrically revise and revalidate the three core dimensions of the Five-Dimensional Altered States of Consciousness Rating Scale (5D-ASC). We aim to investigate whether a coherent structure between higher-order factors (dimensions; Dittrich et al., 2006, 2010) and lower-order factors (subscales; Studerus et al., 2010) can be achieved using a large dataset including several classic psychedelics across a wide dose range. While this Introduction focuses on the historical development of the ASC lineage to motivate the present revalidation, other instruments are also widely used to assess psychedelic experiences rather comprehensively—notably the Psychedelic Experience Scale/Mystical Experience Questionnaire (PES/MEQ; Barrett et al., 2015; Griffiths et al., 2006; MacLean et al., 2012; Pahnke, 1963; Richards, 1975; Stocker et al., 2024) and the Hallucinogen Rating Scale (HRS; Calder et al., 2024; Strassman et al., 1994), alongside more targeted tools such as the Emotional Breakthrough Inventory (EBI; Roseman et al., 2019), the Psychological Insight Questionnaire (PIQ; Davis et al., 2021), the Challenging Experience Questionnaire (CEQ; Barrett et al., 2016), the Ego Dissolution Inventory (EDI; Nour et al., 2016), and the Watts Connectedness Scale (WCS; Watts et al., 2022). A comparative appraisal of the broader instruments (ASC, PES/MEQ, HRS) and how the more targeted tools (EBI, etc.) fit into the picture is provided in the “Discussion.”

The 5D-ASC is a widely used psychometric tool to measure non-ordinary states of consciousness (NSCs).^1^ The 5D-ASC was the result of decades of psychometric research on NSCs initiated and led by Adolf Dittrich (1975, 1985, 1996, 1998; Dittrich et al., 2006, 2010). His earliest scientific engagement with NSCs can be traced back to the early 1970s with a controlled single-subject study at the Psychiatric University Clinic Zurich. Under double-blind conditions, he was administered low doses of mescaline, with and without pretreatment using a structurally related phenethylamine, to examine potential agonist-antagonist interactions. In a subsequent open trial, he ingested 250 mg of mescaline to explore the effects of a somewhat higher dose (Dittrich, 1971).

In 1975, Dittrich published the Abnorme / Aussergewöhnliche Psychische Zustände (APZ),^2^ a precursor to what would later become the 5D-ASC. For the APZ, Dittrich drew on a wide range of sources, including questionnaires, observer ratings, clinical descriptions, own clinical experience, and self-experiments with psychoactive substances. This process yielded approximately 800 items, which he systematically reduced to 158. The first major APZ milestone came in 1985, when after a decade of research, Dittrich (1985) published the first psychometric findings. Using the previously developed APZ scale (Dittrich, (1975), Dittrich (1985) demonstrated that NSCs, could be captured through three primary dimensions that he named: Oceanic Boundlessness (OB), Anxious Ego Dissolution (AED), and Visionary Restructuralization (VR), and that these were independent of etiology—whether induced pharmacologically, psychologically, or spontaneously. These three dimensions (49 items) then also formed the basis of a still broader, etiology-independent 72-item ASC scale within the APZ.

The next major APZ revision, which also led to a name change of the scale, came in the late 1980s. The OB dimension was adapted to more fully capture characteristics of mystical experience as described by Stace (1960), while the VR dimension was expanded with items related to imagination, originality, and memory, following Leuner (1981). Given the initials of the three core dimensions—OB, AED, and VR—this revised 70-item version was named *OAV* (Bodmer, 1989; Bodmer et al., 1994). Hereafter, we refer to the OAV as the three-dimensional ASC (3D-ASC) to make it transparent that this aggregate stands for the three core dimensions of the 5D-ASC—in line with recent terminology of our lab, including collaborations with other labs (Angerer et al., 2024; Arikci et al., 2025; Becker et al., 2025; Gasser et al., 2025; Holze et al., 2023).

A further developmental step for the 3D-ASC was its integration into the 5D-ASC: the 3D-ASC (OAV) was combined with two further validated dimensions—auditory alterations (AA) and vigilance reduction (VIR)—to create the five-dimensional 5D-ASC as we know it today (Dittrich et al., 2006, 2010). As AA and VIR were not primarily designed for classic (serotonergic) psychedelics—but rather, for example, to capture auditory effects in sensory deprivation and hypnagogia (AA), and the effects of dissociatives and sedatives (VIR; Dittrich et al., 2006, 2010)—they are not included in the current analysis. We focus instead on the 3D-ASC dimensions, which in the 5D-ASC comprise 66 items.

A turning point for the 3D-ASC (OAV) came in 2010: Studerus et al. published a psychometric reevaluation of the 3D-ASC part of the 5D-ASC using data from pharmacologically induced NSCs (psilocybin, ketamine, and methylenedioxymethamphetamine (MDMA)). This analysis deconstructed Dittrich’s original 66-item, three-dimensional 3D-ASC structure (which had no subscales) into a 42-item structure comprising 11 subscales (Studerus et al., 2010). This model provided a superior fit and allowed for more fine-grained analyses of specific experiential components than Dittrich’s original model. However, the novel model no longer included any higher-order dimensions. While Studerus et al. (2010) emphasized the theoretical utility of both broad and narrow-band constructs depending on their intended use (p. 15)—predicting, respectively, heterogeneous/complex criteria versus more homogeneous/specific criteria—they did not find sufficient empirical support for Dittrich’s three-dimensional 3D-ASC structure. Nevertheless, despite the findings of Studerus et al., many researchers continue to report their findings using Dittrich’s original three-dimensional 3D-ASC structure, presumably mainly due to its greater suitability for summarizing broader experiential domains.

This left the field with a dilemma: while the 5D-ASC can either be analyzed with the original *3D-ASC (OAV) scale*, based on Dittrich’s 66 items and three higher-order dimensions (Dittrich et al., 2006, 2010) or with *the eleven-subscale ASC (11-ASC*^3^*) scale*, based on Studerus et al.’s (2010) 42 items and 11 lower-order subscales, the factorial structure of the two cannot be reconciled. Table 1 summarizes the main revisions from the APZ to the OAV/3D-ASC, its integration into the 5D-ASC, and the 11-ASC reanalysis, clarifying the naming conventions and factor-analytic changes across versions.

**Table 1.** Key stages in the development of the ASC scales.

Year	Scale	Main features	Notes
1975	APZ	Initial development (~800 items reduced to 158)	Dittrich drew on questionnaires, observer ratings, clinical descriptions, self-experiments
1985	APZ (3D model)	First large analysis of APZ, which resulted in three dimensions: OB, AED, VR (APZ 3D: 49 items in total)	Demonstrated etiology-independence (pharmacological, psychological, spontaneous)
1989	OAV/3D-ASC	Revision of the three dimensions of the APZ, enriching OB (mystical) and VR (imagination, originality, memory) (OAV: 70 items in total)	OAV acronym from OB, AED, VR; more recently, also referred to as the 3D-ASC
2006/2010	5D-ASC	Integration of 3D-ASC (OAV) with AA and VIR to form 5D-ASC (OAV part: 66 items in total)	AA, VIR; not primarily psychedelic-relevant
2010	11-ASC	Reanalysis of OAV part of the 5D-ASC resulting in 11 subscales (11-ASC) with no longer any dimensions (11-ASC: 42 items in total)	3D-ASC and 11-ASC factorial structures irreconcilable

ASC: Altered States of Consciousness Rating Scale; APZ: Abnorme / Aussergewöhnliche Psychische Zustände; OB: Oceanic boundlessness; AED: Anxious ego dissolution; VR: Visionary restructuralization; OAV: acronym from OB, AED, VR; AA: Auditory alterations; VIR: Vigilance reduction.

Contemporarily, some researchers only report Dittrich’s 3D-ASC analysis (Griffiths et al., 2016; Passie et al., 2021), others rely solely on Studerus’ 11-ASC analysis (Huels et al., 2021; Sumner et al., 2021), and many report both without a coherent factor structure linking them (Becker et al., 2022a; Holze et al., 2021; Hovmand et al., 2024b; Preller and Vollenweider, 2018; Smigielski et al., 2019). This divergence in analytic approaches across international research groups hampers meaningful comparison and integrative interpretation of findings. While Studerus et al. note that broader dimensions are typically better suited to predict heterogeneous or complex outcomes, and narrower constructs (subscales, facets) are more appropriate for specific criteria (Reise et al., 2000; Studerus et al., 2010: 15–16), these complementary levels of analysis ideally require a coherent hierarchical structure. Our main hypothesis is that the 42-item 11-ASC of Studerus et al. can be factorially integrated into a 3D-ASC-like (OAV-like) three-dimensional structure, restoring conceptual continuity and interpretive clarity, while also preserving the improved psychometric resolution.

These three higher-order dimensions are conceptually derived from Dittrich’s original dimensions (Dittrich et al., 2006, 2010) OB, AED, and VR. For greater clarity and accessibility, we relabel them as *Positive Effects (PosE)*, encompassing usually positively valenced and meaningful experiences that can, at their strongest form, take on mystical or transcendent qualities. The inclusion of mystical and transcendent qualities (unitive, spiritual, and body-boundary transcending qualities) under the umbrella term “PosE” corresponds to the notion that such experiences are often described as blissful (Eckhart, 1995: 29 [Sermon 2], 2009: 79–80 [Sermon 8]; Otto, 1926: 45, 1932: 34; Stace, 1960: 131–133). *Distressing Effects (DisE)* refers to usually negatively valenced states such as anxiety, alienation, and disruptions in thought and volition. Finally, *Perceptual Effects (PerE)* captures phenomena such as simple and complex visuals and audiovisual synesthesia. These revised labels preserve theoretical continuity while offering more contemporary, transparent, and user-friendly terminology.

We refer to the proposed integration as the *3D-ASCr* scale, with “3D” denoting three higher-order dimensions and “*r*” standing for *revised*. We further hypothesize that this 42-item structure will show better model fit than Dittrich’s original 66-item, three-dimensional 3D-ASC model, aligning with findings by Studerus et al. (2010).

More recently, Hovmand et al. (2024a) and Rodrigues et al. (2025) also reported superior model fit for the 11-ASC compared to Dittrich’s original 3D-ASC model—based on psilocybin data from largely naturalistic settings in the case of Hovmand et al. (2024a), and on naturalistic data with classic psychedelics, ketamine, MDMA, and other substances in the case of Rodrigues et al. (2025). However, like Studerus et al. and other studies to date, the analyses of these two studies did not assess whether the eleven subscales could be integrated into a coherent higher-order structure. The dimensional and subscale models were evaluated side by side, but the possibility of unifying them into a single hierarchical architecture remained unexplored.

Overall then, the 11-ASC has shown robust and acceptable model fit, while the 3D-ASC has not (Hovmand et al., 2024b; Rodrigues et al., 2025; Studerus et al., 2010). Because of this established evidence—and also to allow for continuity and comparability with already gathered data in the field—we reasoned that it is desirable to retain the 11-ASC structure rather than to return to item-level analyses. Yet, given the value of the 3D-ASC’s conceptual clarity and conciseness, we decided to try to combine the advantages of both models—11-ASC and 3D-ASC—in our 3D-ASCr model. Thus, the aim of the present analysis is to reevaluate the dimensions and subscales of the 3D-ASC by applying exploratory and confirmatory factor analyses (EFA and CFA) to the 42-item 11-ASC of Studerus et al.(2010) in the context of the proposed 3D-ASCr structure. In line with these aims, we hypothesize that the 11 subscales of the 11-ASC can be factorially integrated into a 3D-ASC-like (OAV-like) three-dimensional structure, and that furthermore this 42-item structure will show better model fit than Dittrich’s original 66-item, three-dimensional 3D-ASC model, aligning with findings by Studerus et al. (2010), Hovmand et al. (2024a), and Rodrigues et al. (2025). We conduct these analyses within a large, controlled laboratory dataset including several classic psychedelics across a wide dose range. Compared to earlier evaluations also conducted under controlled laboratory conditions (Dittrich, 1985; Dittrich and Lamparter, 1991b (as cited in Dittrich et al., 2006, 2010); Hovmand et al., 2024a; Studerus et al., 2010), the present study advances the field in three key respects: (1) it includes the largest classic-psychedelic dataset to date, with 901 completed 5D-ASC questionnaires (vs. 327 in Studerus et al.); (2) it spans a wider range of classic psychedelics including lysergic acid diethylamide (LSD), psilocybin, mescaline, and N,N-dimethyltryptamine (DMT; vs. a maximum of two substances in previous studies); and (3) it covers a broader dose range, from low to very high doses (whereas prior studies ranged at most from low to high, or did not vary doses). Together, these extensions provide a strong empirical basis for testing the proposed 3D-ASCr structure.

## Methods

### Participants

A total of 901 5D-ASC questionnaires from 16 different randomized and mostly placebo-controlled classic-psychedelic studies using LSD, psilocybin, mescaline, or DMT from an overall number of 398 healthy participants (mean age = 32.6, SD = 9.4, ranging from 25 to 65; 203 females) were included for the current analysis (Arikci et al., 2025; Becker et al., 2022a, 2022b, 2025; Erne et al., 2024; Holze et al., 2020, 2021, 2022; Klaiber et al., 2024; Ley et al., 2023; Liechti et al., 2017; Schmid et al., 2015; Straumann et al., 2023; Vogt et al., 2023; NCT05523401; NCT05695495). To maximize statistical power, we deliberately pooled data from classic 5-HT_2A_ agonist psychedelics (LSD, psilocybin, mescaline, DMT), which share highly similar profiles of NSCs (Erne et al., 2024; Holze et al., 2022; Ley et al., 2023). We included the use of the 5D-ASC with psychedelic doses ranging from very high to low (see Supplemental Material 1 for details). We did not consider 5D-ASC data if very low doses (25 µg LSD, 100 mg mescaline, 5 mg DMT) were used, or if the serotonergic psychedelics were co-administered with another drug (MDMA, ketanserin, escitalopram, paroxetine), or if the dose varied individually (self-guided titration for DMT).

### Material

#### Five Dimensions of Altered States of Consciousness (5D(11)-ASC) scale

Psychedelic experience was assessed using the original German version of the 5D-ASC with 94 items (visual analog scales ranging from 0 to 100; Dittrich, 1998; Dittrich et al., 2006). Currently, the instrument is validated for five higher-order factors (dimensions) using all 94 items (Dittrich et al., 2006, 2010) or for 11 lower-order factors (subscales) using 42 items (Hovmand et al., 2024a; Rodrigues et al., 2025; Studerus et al., 2010), but, factorially speaking, the higher-order factors and the lower-order factors do not form a coherent whole (see “Introduction” and “Discussion” for a description of the current higher-order and lower-order factors of the 5D(11)-ASC)).

#### Data analysis

In a first step, we evaluated and compared the following two basic analytical models in terms of reliability and validity: (1) the original 66-item 3D-ASC model with the three dimensions (which have no subscales) OB, AED, VR from Dittrich et al. (2006, 2010), and (2) the 42-item 11-ASC model with 11 subscales (which have no dimensions) from Studerus et al. (2010). The reliability of the subscales was evaluated using measures of internal consistency, Cronbach’s α, and McDonald’s ω, and the validity of the factor structure was assessed through model fit indices from CFA. In parts of one DMT study (NCT05695495; *n* = 113), only the 42 items of the 11-ASC were administered, which did not allow computation of the 3D-ASC model. To enable fair comparison with the 11-ASC model, we also excluded these data for the CFA of the 11-D ASC.

In a second step, we explored whether the 11 subscales from Studerus et al. (2010) could be integrated into a coherent higher-order structure. To this end, we conducted an EFA (maximum likelihood extraction, oblimin rotation) with the 11 subscale means with half of the data, and used the other half to run a CFA on the solution suggested by the EFA. To split the data in half, each participant was randomly allocated either to the exploratory or confirmatory data set within each study. The EFA was performed using principal axis factoring, which is recommended when the assumption of multivariate normality may be violated (Costello and Osborne, 2005; Fabrigar et al., 1999). An oblimin rotation was applied because the underlying factors were expected to be correlated (Hayton et al., 2004). This combination of principal axis factoring and oblimin rotation has previously been employed in the validation of the 5D-ASC (Rodrigues et al., 2025).

As model fit indices in the CFAs, we assessed normed Chi-Square χ^2^/df, which evaluates the discrepancy between the observed and expected covariance matrices relative to the model’s degrees of freedom, the root mean square error of approximation (RMSEA), which estimates how well the model approximates the data while accounting for model complexity, the standardized root mean square residual (SRMR), which reflects the average standardized difference between observed and predicted correlations, and the comparative fit index (CFI), which compares the fit of the proposed model to a null model (no relationships between variables), adjusting for sample size. Fit indices of the CFA are based on robust maximum likelihood estimation with Satorra-Bentler correction (see Stocker et al., 2024). Results were interpreted following the conventional cutoff criteria for model fit: χ^2^/df < 2, RMSEA < 0.06, SRMR < 0.08, and CFI > 0.95 for good fit; and χ^2^/df < 3, RMSEA < 0.10, SRMR < 0.10, and CFI > 0.90 for acceptable fit (Hu and Bentler, 1999; Tabachnick and Fidell, 2007).

All analyses were conducted in R (R Core Team, 2023) using RStudio (Posit Team, 2025). Cronbach’s α, and McDonald’s ω were calculated with the *psych* package (Revelle, 2023), and factor analyses were performed with the *lavaan* package (Rosseel, 2012).

## Results

Means, SD, and reliability measurements for each dimension/subscale (Dittrich’s 3D-ASC dimensions and Studerus’ 11-ASC subscales) are summarized in Table 2. All measures show acceptable-to-high reliability of the subscales; all α and ω values (Hair et al., 2010; Viladrich et al., 2017). An extended Table of dimensions/subscale means split by dose levels can be found in Supplemental Material 5.

**Table 2.** Means, standard deviations, and reliability indices (Cronbach’s α, McDonald’s ω) for the 3D-ASC dimensions and 11-ASC subscales.

Dimensions/subscales	Items *N*	*M* (SD)	Cronbach’s α	McDonald’s ω
3D-ASC				
OB	27	36.9 (25.0)	0.96 [0.96, 0.96]	0.97
AED	21	16.3 (17.3)	0.92 [0.91, 0.93]	0.94
VR	18	41.4 (23.7)	0.92 [0.91, 0.93]	0.94
11-ASC				
Experience of unity	5	36.3 (31.1)	0.89 [0.88, 0.90]	0.91
Spiritual experience	3	23.0 (25.9)	0.76 [0.74, 0.79]	0.77
Blissful state	3	42.5 (32.8)	0.89 [0.88, 0.90]	0.89
Insightfulness	3	29.5 (28.5)	0.83 [0.81, 0.84]	0.83
Disembodiment	3	33.9 (32.4)	0.86 [0.85, 0.88]	0.87
Impaired control and cognition	7	24.5 (22.6)	0.85 [0.83, 0.86]	0.89
Anxiety	6	9.1 (18.0)	0.91 [0.90, 0.92]	0.94
Complex imagery	3	46.0 (33.0)	0.80 [0.78, 0.82]	0.80
Elementary imagery	3	57.5 (33.7)	0.85 [0.83, 0.87]	0.87
Audiovisual synesthesia	3	51.7 (37.0)	0.93 [0.93, 0.94]	0.94
Changed meaning	3	27.8 (26.6)	0.76 [0.73, 0.78]	0.76

ASC: Altered States of Consciousness Rating Scale; OB: Oceanic boundlessness; AED: Anxious ego dissolution; VR: Visionary restructuralization; SD: Standard deviation.

Results of the CFA are summarized in Table 3.

**Table 3.** CFA model fit indices for the 3D-ASC and 11-ASC.

Analysis	Npar, obs	χ^2^/df	CFI	RMSEA	SRMR
3D-ASC	135, 772	8822.11/2076 = 4.25	0.72	0.076	0.104
11-ASC	139, 774	1766.83/764 = 2.31	0.93	0.049	0.049

*Note.* All fit indices are based on robust maximum likelihood estimation with Satorra-Bentler correction.

ASC: Altered States of Consciousness Rating Scale; CFA: Confirmatory factor analysis; Npar: Number of parameters estimated by the model; obs: Number of observations; χ^2^/df: Normed Chi-Square; CFI: Comparative fit index; RMSEA: Root mean square error of approximation; SRMR: Standardized root mean square residual.

The three-dimensional (subscale-free) 3D-ASC model showed overall poor fit to the data (χ²/df = 4.25, robust CFI = 0.72, robust RMSEA = 0.076, robust SRMR = 0.104). In contrast, the 11-subscale (dimension-free) 11-ASC model demonstrated good fit across all indices (χ²/df = 2.31, robust CFI = 0.93, robust RMSEA = 0.049, robust SRMR = 0.049). These results support the finer-grained 11-ASC subscale structure as a more adequate representation of the data, despite its greater complexity, and imply that summing all 66 items to the three factors OB, AED, VR is not a valid approach, despite common practice. Nonetheless, this widespread practice underscores a persistent demand for a more tractable and conceptually coherent summary of the data. In order to meet this need, we aim to examine whether the 11 subscales can be meaningfully organized into broader, higher-order 3D-ASC-like dimensions.

### Higher-order structure in the 11 subscales

#### Exploratory factor analysis

Based on parallel analysis, the EFA suggests four factors with the following loadings for each subscale (Table 4). Scree plot and Eigenvalues can be found in the Supplemental Material 5. We also considered other criteria: Velicer’s MAP (Velicer, 1976) suggested two factors (minimum average squared partial correlation = 0.05), and Very Simple Structure (Revelle and Rocklin, 1979) pointed to three factors (maximum fit = 0.93). Given that parallel analysis is widely considered the most robust criterion (Hayton et al., 2004), we retained four factors for the EFA.

**Table 4.** Factor loadings from EFA of the 11 ASC subscales.

Subscale	Factor			
	1	2	3	4
Experience of unity	0.84			
Spiritual experience	0.81			
Blissful state	0.80			
Insightfulness	0.83			−0.35
Disembodiment	0.43			0.33
Impaired control and cognition			0.86	
Anxiety			0.77	
Complex imagery		0.57		
Elementary imagery		0.78		
Audio-visual synesthesia		0.90		
Changed meaning	0.37			

*Note.* Values represent factor loadings. Values smaller than 0.3 are omitted.

EFA: Exploratory factor analysis; ASC: Altered States of Consciousness Rating Scale.

The subscales *Experience of Unity*, *Spiritual Experience*, *Blissful State*, and *Insightfulness* loaded strongly on Factor 1. *Complex Imagery*, *Elementary Imagery*, and *Audio-Visual Synesthesia* showed moderate to high loadings on Factor 2, while *Impaired Control and Cognition* and *Anxiety* loaded strongly on Factor 3. *Disembodiment* exhibited cross-loadings above 0.30 on multiple factors, with their highest loading on Factor 1. Lastly, *Changed Meaning* loaded low-to-moderate on Factor 1. Factor 4 was defined by only two subscales, both of which exhibited higher primary loadings on other factors. Given the minimal and cross-loading structure, Factor 4 did not represent a distinct or conceptually meaningful construct and was therefore considered uninterpretable and omitted from further consideration.

Based on this loading pattern, we propose the following three-factor solution with the following names (cf. Introduction):

- Factor 1: *PosE*—includes *Experience of Unity*, *Spiritual Experience*, *Blissful State*, *Insightfulness*, *Disembodiment*, and *Changed Meaning*
- Factor 2: *DisE—*consists of *Impaired Control and Cognition* and *Anxiety*
- Factor 3: *PerE*—comprises *Complex Imagery*, *Elementary Imagery*, and *Audio-Visual Synesthesia*

#### Confirmatory factor analysis

It was not possible to fit the hierarchical three-dimensional model with *DisE* as a higher-order factor in the CFA. This limitation arose because the factor was defined by only two first-order constructs (*Anxiety* and *Impaired Cognition/Control*), which is generally considered insufficient for proper identification and stability of latent variables in CFA models (Brown, 2015). Furthermore, the *Anxiety* subscale exhibited a low mean restricted variance, with 42% of all responses at the floor value (zero), which led to a negative residual variance estimation. Given these issues, we excluded *Anxiety* from the model and retained *Impaired Cognition and Control* as the sole indicator of the higher-order construct *DisE*.

**Figure 1.**
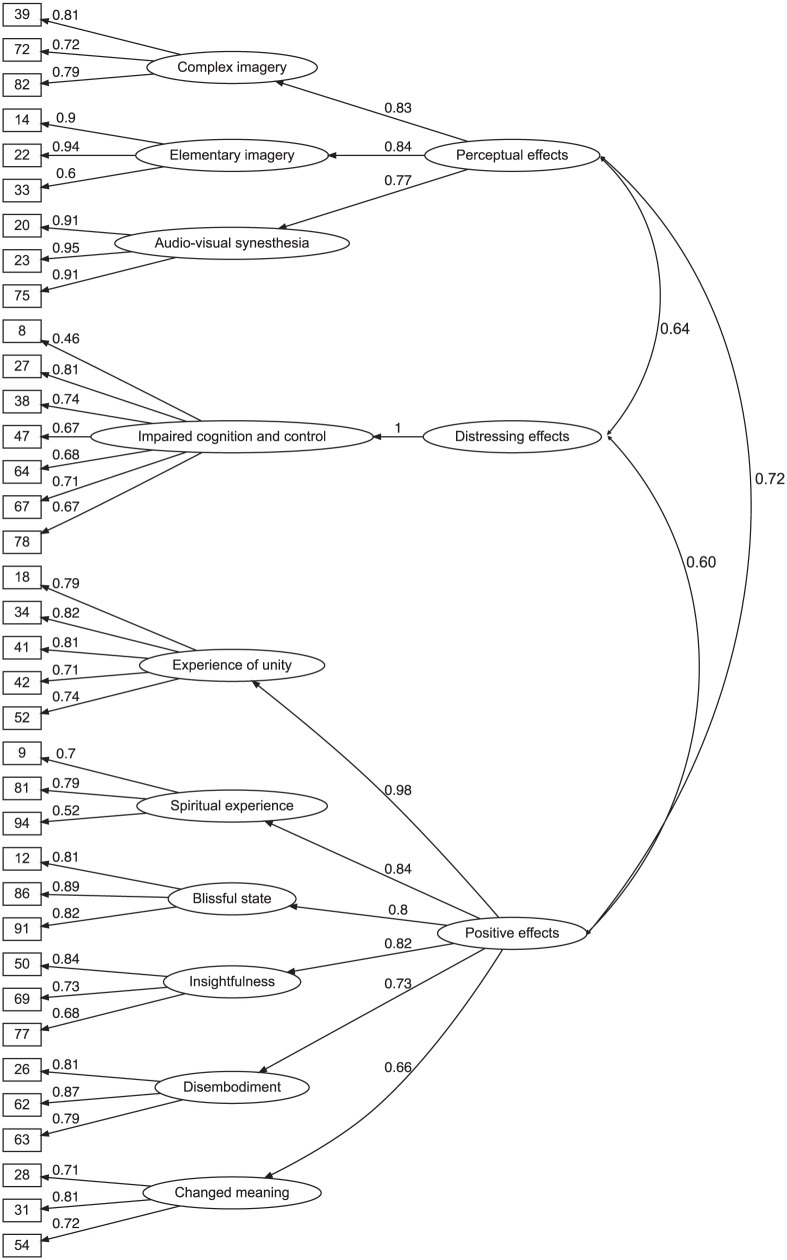
Result of the CFA. Standardized loadings are shown on single-headed arrows, and covariances between higher-order factors are depicted with double-headed arrows. The numbers on the far left represent the item numbers of the 94-item 5D-ASC. ASC: Altered States of Consciousness Rating Scale; CFA: Confirmatory factor analysis.

The model demonstrated acceptable overall fit to the data, with χ²/df ratio = 2.50, robust RMSEA = 0.067, robust SRMR = 0.077, all falling within commonly accepted thresholds. The robust CFI was 0.89, falling just below the conventional cutoff for acceptable fit of 0.90, suggesting margin but tolerable fit. As shown in Figure 1, most item-level loadings are well above 0.70 (strong), again confirming good representation of their respective latent constructs as suggested by Studerus et al. (2010). Most importantly, second-order loadings for PosE und PerE ranged from 0.66 to 0.98, indicating that the first-order factors are well accounted for by the higher-order dimensions. The higher-order factors were moderately correlated, with correlations ranging from 0.60 to 0.72, supporting related but distinct dimensions. Overall, the results of the CFA suggest that the hierarchical three-factor structure is a plausible approximation of the data.

It is important to note that the three newly proposed higher-order factors closely align with Dittrich’s original three 3D-ASC (OAV) dimensions. This is reflected in the very high pairwise correlations between the corresponding concepts: PosE and OB (Spearman’s ρ = 0.98, *p* < 0.001), PerE and VR (Spearman’s ρ = 0.95, *p* < 0.001), and DisE and AED (Spearman’s ρ = 0.98 when Anxiety was included in the DisE mean, and Spearman’s ρ = 0.94 when Anxiety was excluded; both *p*s < 0.001).

## Discussion

We first situate our findings within the ASC questionnaire family, and then place them in the broader context of other existing psychometric measures of psychedelic experiences. Our main hypothesis—that the 11 subscales from the 42-item *11-ASC* (Studerus et al., 2010) can be established not only as distinct lower-order domains but also as forming three higher-order dimensions (*PosE*, *DisE*, *PerE*) akin to *3D-ASC (OAV;* Dittrich et al., 2006, 2010)—has been confirmed for 10 of the 11 subscales. As this hypothesis provides the rationale for the label *3D-ASCr*, we propose *3D-ASCr* as the name of the revised scale. Only the subscale *Anxiety* could not be integrated into *DisE*, primarily due to restricted variance and floor effects that led to improper model estimation and instability in the confirmatory analysis. That said, it could be argued that it is a positive finding that overall levels of anxiety were low in persons administered with classic psychedelics. It seems plausible that, in general, greater anxiety only emerges with sufficient intensity at higher doses to be reliably captured and modeled. Consistent with this, our results show that anxiety increases with dose level, but it remains markedly lower in magnitude compared to the other subscales (see Supplemental Material 5). Nevertheless, we strongly recommend *retaining* the *Anxiety* subscale within the 3D-ASCr, as it captures a phenomenon of theoretical and clinical relevance. Based on content relatedness and results from our EFA, and depending on the dataset and research purpose, it may either be included in DisE or reported as a standalone subscale (see Supplemental Material 4, 3D-ASCr Scoring Key).

Our complementary hypothesis that the 42-item *3D-ASCr* will show better model fit than the 66-item original three-dimensional (subscale-free) structure of Dittrich’s 3D-ASC was also confirmed. In practical terms, this means that it is no longer psychometrically valid to sum individual items into Dittrich’s original three dimensions. Instead, researchers and practitioners should first compute the mean scores of the 11 subscales, and then derive the three higher-order factors from those. This revised approach yields scores that closely approximate the original 3D-ASC dimensions, but with greater psychometric validity, fewer items, a clearer structure, and more intuitive labels—thereby facilitating interpretation and communication of findings. Yet, the fact that our three newly proposed dimensions (*PosE*, *DisE*, *PerE*) strongly resemble Dittrich’s original three 3D-ASC (OAV) dimensions (*OB*, *AED*, *VR*), as shown by high pairwise correlations between the two, stands as a clear testament to the enduring validity of Dittrich’s vision that such a threefold framework can meaningfully capture the core phenomenological dimensions of NSCs (Dittrich, 1985, 1996, 1998; Dittrich et al., 2006, 2010).

The reduction from Dittrich’s original 66-item, three-dimensional 3D-ASC (Dittrich et al., 2006, 2010) to Studerus’ 42-item, 11-ASC subscale structure (Studerus et al., 2010)—now mirrored in our revalidation of the 5D-ASC core dimensions from OB to PosE, from AED to DisE, and from VR to PerE—results in a loss of certain content. We will now briefly discuss the basic content of our three validated dimensions—as well as the omissions and consider how substantial they are for each of the three dimensions.

*PosE* contains the six validated subscales *Experience of Unity*, *Spiritual Experience*, *Blissful State*, *Insightfulness*, *Disembodiment*, and *Changed Meaning*,^4^ from Studerus et al. (2010). All items that make up the first five subscales originate from Dittrich’s original OB dimension (Dittrich et al., 2006, 2010) but with one exception: Dittrich et al. (2010) included original thought (“I had very original thoughts,” #77^5^) under VR, but we follow Studerus et al. (2010) in assigning it to the subscale *Insightfulness*, which otherwise consists of items from OB. Generally, these subscales conceptually align well with Dittrich’s original basic vision of OB—namely that “this . . . happiness-inducing . . . experience . . . in its extreme form . . . can also be experienced as a mystical . . . experience” (Dittrich et al., 2010: 9). The items of the sixth subscale, *Changed Meaning*, however, stem from Dittrich’s original VR dimension—the dimension that has now become PerE in our analysis. Yet, given Dittrich’s original vision of OB as also in its extreme form including mystical experience, it may not be surprising that the items of this subscale (e.g. “Some everyday things gained a special meaning,” #28) align well with PosE. One example of such a meaning-enhancing psychedelic experience leaning toward the mystical is described by Aldous Huxley (1954), when he recounts perceiving the “divine source of all existence” in “a bunch of flowers” (p. 5).

Two *positive* experiential themes that are notably *reduced or lost* in the revalidation from OB to PosE are mystical-like beauty (loss of “Many things appeared breathtakingly beautiful to me,” #57) and an altered sense of time and space (e.g. loss of “My sense of time and space was altered, as if in a dream,” #36). As long as the construct of PosE remains aligned with Dittrich’s broader conceptualization of OB as encompassing the potential for mystical experience (Dittrich et al., 2010: 9), the omission of a deep, possibly subject-object-transcending sense of beauty is not trivial. Many scholars view beauty as an essential characteristic of mystical experience—or at least as inextricably intertwined with it (Barwell, 1937: 122; De Villiers, 2016; Richards, 2015: 52–54; Underhill, 2018: 15; cf. also James, 1902: 396; Maslow, 1964: 121–123)—a view that has also found recent psychometric support (Stocker et al., 2024). In contrast, the loss of explicit references to altered time and space may be less critical from a mystical perspective, as one of the most transcendent expressions of this theme, a sense of timelessness that may also imply spacelessness, is still represented in the PosE subscale *Experience of Unity* with the item “I sensed a touch of eternity” (#41). Further notable losses resulting from the revalidation from OB to PosE are pleasurable bodily sensations (#3), feelings of transformation (#16), and feeling an extraordinary power within oneself (#40).

*DisE* contains the seven-item validated subscale *Impaired Control and Cognition* from Studerus et al. (2010), whose main themes, as the name implies, are *impaired volition* (e.g. “I had the feeling that I no longer had a will of my own,” #78) and *impaired thought* (e.g. “My thoughts kept breaking off; I could not think anything through to the end,” #67). All items from this subscale originate from Dittrich’s original AED dimension. Notably, however, this subscale, and therefore DisE more broadly, also includes a distinct experiential theme not reflected in the subscale’s label: *isolation*, as captured by this item: “I felt isolated from everything and everyone” (#64). This aligns with psychometric findings from Stocker et al. (2024), which identified isolation as a typical feature of distressing psychedelic experiences.

Two distressing themes in particular that are *no longer explicitly represented* in the revalidation from AED to DisE are: *suffering*, as in “I felt tormented” (#21), and *bodily disintegration*, as in “My body felt numb, lifeless and strange,” (#60). The loss of suffering is not trivial, as Stocker et al. (2024) show that this be an inherent part of the validated factor Distressing Experience of the PES (with the item “Emotional and/or physical suffering”). The loss of bodily disintegration (#60) may be less consequential for a factor-analytic structure, as similar disintegration-related items also failed to load onto the distressing factor in this PES analysis by Stocker et al. (2024). While the theme of disintegration, which is often followed by a sense of renewal, is widely reported in psychedelic experiences (Grof and Halifax, 1978: 51–54; Richards, 2015: 60–61), bodily disintegration may represent just one of several distinct manifestations of this theme, alongside experiences such as feeling as if dying or fearing the loss of sanity (Richards, 2015: 60). Given that factor analysis presupposes *and-relations* among items—that is, items within a factor are assumed to co-occur—a disintegration construct may be poorly suited to this logic. It may be better conceptualized through *or-relations*, in which one might undergo a death experience, *or* a fear-of-going-insane experience, *or* a bodily disintegration experience, without all necessarily occurring within the same individual session, as factor logic would require.

That the 11-ASC subscale *Anxiety* did not load onto the distressing dimension is likely due, as mentioned, to its overall low occurrence in the sample. However, given that the subscale performs well psychometrically as a standalone measure, and given its theoretical and clinical relevance, we, as mentioned, strongly recommend including it when applying the 3D-ASCr scale (see Supplemental Material 3, Scoring Key). It is nonetheless noteworthy that in the validation of the PES (Stocker et al., 2024)—conducted on many of the same participants and sessions—we were able to establish a generic fear item (“Experience of fear”) as part of a validated distressing factor. One possible explanation for this discrepancy may lie in the level of specificity: while the PES item is phrased broadly, the 11-ASC *Anxiety* subscale includes more specific expressions of fear or unease, such as “I was afraid I wouldn’t be able to get out of the state I was in”(#32), “I was afraid without being able to say exactly why” (#43), or “I felt threatened”(#56). As a result, certain forms of fear—for instance those that may manifest in highly situational, intense, or less easily specified ways—might still fall outside the scope of what the 11-ASC *Anxiety* construct captures. Notably (similarly to the theme isolation occurring in the subscale named *Impaired Control and Cognition*), the *Anxiety* subscale also includes a distinct experiential theme not reflected in the subscale’s label: *alienation*, as captured by a number of items in this subscale, for instance: “I experienced my surroundings as strange and unsettling” (#46). Overall, our new dimension DisE—even when considered alongside the standalone subscale *Anxiety*—somewhat departs from Dittrich’s original basic vision of AED, in which “anxiety is a central aspect . . . [which] can coincide with a feeling of one’s own disintegration or separation from oneself and the world” (Dittrich et al., 2010: 9). In our large sample, anxiety did not emerge as a central feature, but rather as a marginal one, and disintegration may be inherently difficult to capture within a factor-analytic framework. Nevertheless, it remains one of Dittrich’s key contributions to this distressing dimension that he formulated items on impaired volition, impaired thought, and isolation as integral parts of the broader distressing psychedelic-experiential complex—three themes that are now empirically retained within the newly validated DisE dimension.

*PerE* includes the three validated subscales *Complex Imagery*, *Elementary Imagery*, and *Audio-Visual Synesthesia* from Studerus et al. (2010). All items from these three subscales originate from Dittrich’s original VR dimension. This aligns well with one core aspect of how Dittrich originally conceptualized the perceptual dimension—namely, as involving “phenomena of altered perception . . . [by which] are also meant optical hallucinatory phenomena and synaesthesiae, when for example sounds become visible as images” (Dittrich et al., 2010: 9). It aligns less well, however, with the second core component of Dittrich’s definition: the inclusion of “phenomena of altered . . . meaning” (Dittrich et al., 2010: 9) within this dimension. As our factor analysis revealed and as discussed, the subscale *Changed Meaning* stands somewhat apart, yet is sufficiently associated with PosE to warrant its inclusion there rather than in PerE.

Three experiential themes that Dittrich et al. (2006, 2010) also included in his original VR conceptualization—but which are no longer explicitly covered in the revalidation from VR to Per—are altered size perception (“Things around me seemed smaller or larger,” #83), hilariousness (“Many things seemed incredibly funny to me,” #70), and reality assessment of perceptual content (“I saw things I knew were not real,” #7). However, since Dittrich et al. (2010) did not reference these themes in his core description of the VR dimension (p. 9), it may perhaps be inferred that he considered them less central. Moreover, as we are not aware of any strong associations between these themes and clinically or otherwise relevant outcomes, their omission appears justifiable—at least given the current state of knowledge. That said, should future research reveal consistent links between these experiential facets and relevant outcome variables, their reintegration into the broader conceptualization of PerE may be warranted.

We now place our findings with the 3D-ASCr in the broader landscape of psychometric measures of psychedelic experiences, with particular attention to their clinical relevance. Alongside the 3D-ASCr, two other major multidimensional instruments are available that also aim at capturing vital aspects of the psychedelic experience rather comprehensively: the PES48 (Richards, 1975; Stocker et al., 2024) and the *HRS (Version 4;* Calder et al., 2024; Strassman et al., 1994). Hovmand et al. (2024a), alongside with non-validated visual-analog or Likert scales specifically developed for clinical trials, identified these three scales (and/or precursor forms of them) as the most utilized patient-reported outcome measures in clinical research on classic psychedelics. Each of these three major psychometric instruments captures the three broad experiential domains commonly observed in psychedelic states—and relevant to psychedelic therapy: positively valenced or meaningful experiences (up to the transcendent), distressing experiences, and perceptual phenomena. This tripartite structure is explicitly reflected in the 3D-ASCr (via its *PosE*, *DisE*, and *PerE* dimensions) and in the PES48—through the embedded *MEQ40* and the added *Distressing Experience* and *Visual Experience* subscales. The HRS also encompasses these basic domains, though in a more distributed manner across seven factors. Similarly, the seven heuristic domains of Hovmand et al. (2024a, Table 6) also roughly align with these three basic domains—e.g. their heuristic domain “Subjective Positive Reactions,” and many experiences under the domains “Psychological Experience” and “Mystical or Spiritual Nature” fall into the broader domain of positively valenced or meaningful experiences (up to the transcendent). In addition, the HRS includes an experiential domain not covered by the tripartite structure mentioned above, termed *Somaesthesia* in the HRS—which captures interoceptive and bodily sensations and may represent a distinct bodily-affective dimension relevant for certain meaningful or therapeutic processes. All three tools have recently undergone revalidation (Calder et al., 2024; Stocker et al., 2024; and the 3D-ASCr in this paper) and have shown—in earlier or precursor forms—significant correlations with clinical outcomes. High scores on the *OB* dimension or its related subscales from the 5D-ASC (as now revalidated as the 3D-ASCr) as well as high scores on the MEQ (now embedded in the PES48) have mostly predicted positive treatment responses in depression, anxiety, and addiction (Bogenschutz et al., 2015; Carhart-Harris et al., 2018; Garcia-Romeu et al., 2015; Griffiths et al., 2016; Holze et al., 2023; Roseman et al., 2018; Ross et al., 2016). The HRS, while less frequently used in clinical trials, has also shown predictive utility: its *Perception* subscale correlated with antidepressant response (Palhano-Fontes et al., 2019), and its *Intensity* subscale predicted improvements in alcohol use disorder (Bogenschutz et al., 2015).

Given this choice—the 3D-ASCr, PES48, and HRS—what should a practitioner choose? Based on current evidence, we recommend administering both the 3D-ASCr and the PES48 whenever feasible. These two tools have been more consistently linked to clinically relevant outcomes than the HRS and are also more economical in length (42 items for the 3D-ASCr, 48 for the PES48, versus 88 for the HRS). Moreover, while they cover overlapping ground, each scale also contributes important experiential domains not captured by the other. Notably, these unique facets are not just theoretically distinct but have also demonstrated clinical relevance: for instance, Insightfulness (captured only by the 3D-ASCr) has been linked to therapeutic change in depression (Carhart-Harris et al., 2018), while Ineffability (captured only by the PES48) has predicted positive treatment outcomes in cancer-related anxiety (Ross et al., 2016).

Meanwhile, the HRS also captures experiential themes—either as validated factors or as part of broader validated factors—that are neither addressed by the 3D-ASCr nor the PES48. At the same time, the HRS also leaves out other themes covered by either the 3D-ASCr (e.g. ineffability) or the PES48 (e.g. connectedness to the world and beauty). Themes represented as distinct validated factors in the HRS but absent from the other two tools include *Somaesthesia* (e.g. “Electric/tingling feeling”) and positively valenced *Volition* (e.g. “Able to ‘let go’”). Further noteworthy themes not covered by the 3D-ASCr or PES48—yet incorporated within the HRS’s broader *Meaningfulness* factor—include self-acceptance, forgiveness, emotional range, minor senses, and explicit autobiographical content. While the full PES (PES100), which extends the validated PES48 with 52 additional items, does encompass some of these themes as well (Stocker et al., 2024, Supplemental Material 3), only the HRS has integrated these additional themes into a psychometrically validated structure.

Of particular interest from a clinical perspective is the HRS’s explicit targeting of autobiographical content within the *Meaningfulness* factor with the three items “Memories of childhood,” “Feel like a child,” and “Insights into personal or occupational concerns.” Capturing such autobiographical material may be especially valuable in therapeutic contexts: both early clinical reports and contemporary qualitative studies suggest the potential psychological significance of re-experiencing childhood events (González et al., 2019: 271; Jensen and Ramsay, 1963: 138; Renelli et al., 2020: 440; Watts et al., 2017: 539). Similarly, insights into personal concerns—particularly involving interpersonal relationships—have been documented as central to transformation in psychedelic therapy. For instance, in Belser et al.’s study of patients with cancer-related anxiety, all 13 participants “described remarkable insights or transformations involving a significant personal relationship” (Belser et al., 2017: 361; see also Richards, 2015: 87–88, 99–102 for illustrative examples).

As some of the eight HRS factors, including *Meaningfulness*, are thematically broad, future refinements might consider developing more fine-grained lower-order subscales, such as a dedicated *Autobiographical Processing* subscale nested within the *Meaningfulness* domain. Until such revisions are available, we suggest that clinicians and researchers may wish to complement the 3D-ASCr and PES48 with the HRS when time and resources permit, while keeping in mind that the clinical utility of the HRS (Version 4) is still developing and warrants further empirical validation (Calder et al., 2024: 11). The same applies, to some extent, to the 3D-ASCr and the PES48, though here it concerns primarily their newly validated components rather than their already clinically established parts. In the case of the 3D-ASCr, this concerns the revised three-dimensional structure derived from the established 11-ASC (*PosE*, *DisE*, and *PerE*). For the PES48 (Stocker et al., 2024), it concerns the addition of the new MEQ factors *Connectedness* and *Paradoxicality*—which extend the already clinically established MEQ30 to the MEQ40—as well as the newly introduced factors *Visual Experience* and *Distressing Experience*. In Stocker et al. (2024), Connectedness and Paradoxicality showed particularly strong associations with the mystical core of oneness, in fact stronger than Ineffability, suggesting that restricting measurement to the MEQ30 would omit facets central to the mystical construct.

Additionally, these three broader psychedelic-experience tools (3D-ASCr, PES48, HRS) can—and sometimes should—be complemented with psychedelic-experience instruments that are thematically more specialized depending on the clinical or research focus. Instruments that may serve as such targeted complements include the EBI (Roseman et al., 2019), the PIQ (Davis et al., 2021), the CEQ (Barrett et al., 2016), the EDI (Nour et al., 2016), and the WCS (Watts et al., 2022).

Beyond this, and in line with Dittrich’s original vision for this scale (Dittrich, 1975, 1985, 1996, 1998; Dittrich et al., 2006, 2010), future studies may also explore the utility of the 3D-ASCr for other pharmacologically induced (e.g. dissociative) NSCs as well as non-pharmacologically induced ones. Qualitative reports suggest that the three experiential dimensions of the 3D-ASCr may well be applicable also to non-pharmacologically induced NSCs, for instance, to advanced or intensive meditation (Kornfield, 1979; Van Gordon et al., 2018, 2019), sensory deprivation (Kjellgren et al., 2008), and rhythm-induced trance (Flor-Henry et al., 2017; Kjellgren and Eriksson, 2010).

### Strengths and Limitations

The 3D-ASCr validation benefited from a large, diverse dataset (901 sessions from 398 individuals across 16 studies), including 4 different classic psychedelics and a broad dose range. This supports the stability and generalizability of its factor structure across substances and intensities. Many of its subscales or related 3D-ASC dimensions have demonstrated predictive utility as psychometric biomarkers of therapeutic response (Aaronson et al., 2024; Carhart-Harris et al., 2018; Holze et al., 2023; Roseman et al., 2018). A further conceptual strength is that the 3D-ASCr resolves a long-standing divergence between two competing models of the 5D-ASC—Dittrich’s three-dimensional 3D-ASC (OAV) structure (Dittrich et al., 2006, 2010) and Studerus et al.’s (2010) 11 subscale 11-ASC structure—by reestablishing a coherent higher-order dimensional framework grounded in the lower-order 11-ASC item set. This reconciles prior analytic inconsistencies and allows for more meaningful comparisons across studies and research groups.

Nonetheless, certain limitations apply. The sample consisted of healthy volunteers in controlled research settings, which may limit generalizability to patient populations or real-world therapeutic contexts. Furthermore, while our pooling strategy improves generalizability and statistical power, it remains important for future research with larger substance-specific datasets to examine possible differences between these substances in their psychometric profiles. Moreover, while the 3D-ASCr retains validated components from the 5D(11)-ASC, its revised three-dimensional structure (PosE, DisE, PerE) has not yet been tested in clinical settings or linked directly to treatment outcomes. Nevertheless, the three-dimensional model—with Anxiety either integrated into DisE or treated as a standalone addition—is psychometrically well supported. Further research is needed to establish its prognostic or diagnostic relevance. That said, the scale’s conceptual clarity, compactness, and interpretability may already make it a useful tool for clinical trial design, training, or therapeutic development in applied and translational contexts. In the longer term, given that qualitative work in clinical contexts has pointed to prominent gaps in psychedelic psychometrics—such as personal-relationship insights (Belser et al., 2017) and peak experiences beyond current mystical framings (Gasser et al., 2015)—future work could build on large qualitative databases to develop a new quantitative questionnaire (also to be validated) that is concise yet comprehensive, reducing item number while retaining clinical relevance.

## Conclusion

The present findings suggest that the 3D-ASCr holds strong promise for use with classic (serotonergic) psychedelics in research and clinical practice. The 3D-ASCr provides a multidimensional framework that could help capture the richness and psychological depth of NSCs in a structured and scalable way. We recommend using the presently revalidated and updated 3D-ASCr in future studies. The German and English versions of the 42-item 3D-ASCr are provided in Supplemental Material 2, and the German and English versions of the full original 94-item 5D-ASC in Supplemental Material 3. Both Supplemental Material 2 (42 items relevant for the 3D-ASCr) and Supplemental Material 3 (full 94 items) provide updated English versions of the widely used translation by Hasler and Cahn, incorporating refined linguistic phrasings. These refinements were limited to linguistic adjustments to improve naturalness and closer alignment with the German original. They do not alter the content or psychometric meaning of the items; data previously collected with the Hasler and Cahn version and data collected with the present version remain directly comparable and can be pooled. The same applies to the other existing English translation of the 5D-ASC by Dittrich, Lamparter, and Maurer, which is sometimes also used and is highly similar to the version of Hasler and Cahn. The 3D-ASCr scoring key is available in Supplemental Material 4. This updated scoring and reporting system can also be applied to previously collected ASC data.

## Supplemental Material

sj-docx-1-jop-10.1177_02698811251397328 – Supplemental material for The 3D-ASCr scale: A revalidation of the core dimensions of the Altered States of Consciousness Rating Scale 5D(11)-ASC for psychedelic research Supplemental material, sj-docx-1-jop-10.1177_02698811251397328 for The 3D-ASCr scale: A revalidation of the core dimensions of the Altered States of Consciousness Rating Scale 5D(11)-ASC for psychedelic research by Kurt Stocker, Matthias Hartmann, Yasmin Schmid, Severin B. Vogt, Anna M. Becker, Laura Ley, Isabelle Straumann, Denis Arikci, Aaron Klaiber, Livio Erne, Patrick Vizeli, Friederike Holze and Matthias E. Liechti in Journal of Psychopharmacology

sj-docx-2-jop-10.1177_02698811251397328 – Supplemental material for The 3D-ASCr scale: A revalidation of the core dimensions of the Altered States of Consciousness Rating Scale 5D(11)-ASC for psychedelic research Supplemental material, sj-docx-2-jop-10.1177_02698811251397328 for The 3D-ASCr scale: A revalidation of the core dimensions of the Altered States of Consciousness Rating Scale 5D(11)-ASC for psychedelic research by Kurt Stocker, Matthias Hartmann, Yasmin Schmid, Severin B. Vogt, Anna M. Becker, Laura Ley, Isabelle Straumann, Denis Arikci, Aaron Klaiber, Livio Erne, Patrick Vizeli, Friederike Holze and Matthias E. Liechti in Journal of Psychopharmacology

sj-docx-3-jop-10.1177_02698811251397328 – Supplemental material for The 3D-ASCr scale: A revalidation of the core dimensions of the Altered States of Consciousness Rating Scale 5D(11)-ASC for psychedelic research Supplemental material, sj-docx-3-jop-10.1177_02698811251397328 for The 3D-ASCr scale: A revalidation of the core dimensions of the Altered States of Consciousness Rating Scale 5D(11)-ASC for psychedelic research by Kurt Stocker, Matthias Hartmann, Yasmin Schmid, Severin B. Vogt, Anna M. Becker, Laura Ley, Isabelle Straumann, Denis Arikci, Aaron Klaiber, Livio Erne, Patrick Vizeli, Friederike Holze and Matthias E. Liechti in Journal of Psychopharmacology

sj-docx-4-jop-10.1177_02698811251397328 – Supplemental material for The 3D-ASCr scale: A revalidation of the core dimensions of the Altered States of Consciousness Rating Scale 5D(11)-ASC for psychedelic research Supplemental material, sj-docx-4-jop-10.1177_02698811251397328 for The 3D-ASCr scale: A revalidation of the core dimensions of the Altered States of Consciousness Rating Scale 5D(11)-ASC for psychedelic research by Kurt Stocker, Matthias Hartmann, Yasmin Schmid, Severin B. Vogt, Anna M. Becker, Laura Ley, Isabelle Straumann, Denis Arikci, Aaron Klaiber, Livio Erne, Patrick Vizeli, Friederike Holze and Matthias E. Liechti in Journal of Psychopharmacology

sj-docx-5-jop-10.1177_02698811251397328 – Supplemental material for The 3D-ASCr scale: A revalidation of the core dimensions of the Altered States of Consciousness Rating Scale 5D(11)-ASC for psychedelic research Supplemental material, sj-docx-5-jop-10.1177_02698811251397328 for The 3D-ASCr scale: A revalidation of the core dimensions of the Altered States of Consciousness Rating Scale 5D(11)-ASC for psychedelic research by Kurt Stocker, Matthias Hartmann, Yasmin Schmid, Severin B. Vogt, Anna M. Becker, Laura Ley, Isabelle Straumann, Denis Arikci, Aaron Klaiber, Livio Erne, Patrick Vizeli, Friederike Holze and Matthias E. Liechti in Journal of Psychopharmacology

## Notes

1. We use “NSC” for theoretical neutrality, as it can denote both change in degree and change in kind, whereas “ASC” often implies only the former; we retain “ASC” in the scale name for recognizability.

2. Dittrich (1975) originally introduced the psychometric tool under the name “Abnorme Psychische Zustände” (Abnormal Psychic States), but later redefined the abbreviation as “Aussergewöhnliche Psychische Zustände” (Nonordinary Psychic States/Altered States of Consciousness) to reflect a more neutral and descriptive terminology (Dittrich, 1998).

3. Studerus et al. (2010) did not assign a formal name to their revised 42-item, 11-factor structure. However, some subsequent studies adopted the label “11D-ASC” (McAlpine et al., 2023; Ort et al., 2023; Roseman et al., 2018)—either without specifying what the “D” stands for or explicitly describing the 11 factors as *dimensions*. This usage is conceptually questionable: Studerus and colleagues clearly emphasized that their factors are *lower-order* constructs, and the term dimensions is more commonly reserved for higher-order factors (Reise et al., 2000). We therefore follow the convention of Prugger et al. (2022) and use the more neutral label *11-ASC* to refer to these 11 subscales.

4. While we otherwise retained all original subscale names from Studerus et al. (2010), we shortened *Changed Meaning of Percepts* to *Changed Meaning*, as at least one item (“Some everyday things gained a special meaning,” #28) may—but does not necessarily—relate to perceptual content.

5. These numbers correspond to the item numbers in the 5D-ASC scale (Supplemental Material 3).
