# Supplementary material for: The 3D-ASCr scale: A revalidation of the core dimensions of the Altered States of Consciousness Rating Scale 5D(11)-ASC for psychedelic research
Source: J Psychopharmacol. 2025 Dec 26;40(5):850–62. doi: 10.1177/02698811251397328 (PMC13310268; doi:10.1177/02698811251397328)
Supplement: sj-docx-1-jop-10.1177_02698811251397328 – Supplemental material for The 3D-ASCr scale: A revalidation of the core dimensions of the Altered States of Consciousness Rating Scale 5D(11)-ASC for psychedelic research [file sj-docx-1-jop-10.1177_02698811251397328.docx]

Supplement 1

Table S1. Overview of Studies Included in the 3D-ASCr Validation (N = 901): Substance, Dose Strength, and 5D-ASC Questionnaire Counts

| Dose strength | Substance | Dosing | N (5D-ASC) | Study References |
| --- | --- | --- | --- | --- |
| Very high | LSD base | 200 μg | 64 | (Holze et al., 2021, 2022; Liechti et al., 2017; Schmid et al., 2015) |
|  | DMT | 2.4 mg/min i | 23 | (Erne et al., 2024) |
|  | DMT | 25 mg b + 1.0 mg/min i^1^ | 27 | (Vogt et al., 2023) |
|  | DMT | 25 mg b | 10 | NCT05695495 |
| High | Psilocybin | 30 mg | 28 | (Holze et al., 2022) |
|  | Mescaline | 800 mg | 17 | (Klaiber et al., 2024) |
|  | DMT | 1.8 mg/min | 24 | (Erne et al., 2024) |
|  | DMT | 20 mg b | 32 | NCT05695495 |
| Moderate to high | Psilocybin | 25 mg | 48 | (Becker, Holze, et al., 2022; NCT05523401) |
| Moderate | LSD base | 100 μg | 201 | (Becker et al., 2025; Becker, Klaiber, et al., 2022; Holze et al., 2020, 2021; Ley et al., 2023; Liechti et al., 2017; Straumann et al., 2023) |
|  | Psilocybin | 20 mg | 33 | (Ley et al., 2023) |
|  | Mescaline | 500 mg | 18 | (Ley et al., 2023) |
|  | DMT | 1.2 mg/min i | 23 | (Erne et al., 2024) |
|  | DMT | 15 mg b + 0.6 mg/min i^2^ | 27 | (Vogt et al., 2023) |
|  | DMT | 15 mg b | 35 | NCT05695495 |
| Low to moderate | LSD base | 80 µg | 41 | (Arikci et al., 2025) |
|  | LSD tartrate | 117 µg | 41 | (Arikci et al., 2025) |
|  | Psilocybin | 15 mg | 28 | (Holze et al., 2022) |
|  | Mescaline | 300 mg | 16 | (Ley et al., 2023) |
|  | Mescaline | 400 mg | 16 | (Klaiber et al., 2024) |
|  | DMT | 1.0 mg/min i | 27 | (Vogt et al., 2023) |
| Low | LSD base | 50 μg | 20 | (Holze et al., 2021) |
|  | Mescaline | 200 mg | 16 | (Klaiber et al., 2024) |
|  | DMT | 0.6 mg/min i | 50 | (Erne et al., 2024; Vogt et al., 2023) |
|  | DMT | 10 mg b | 36 | NCT05695495 |

*Note. N = number; b = bolus; i = infusion; ^1^ = bolus dose is very high, while infusion dose is moderate; ^2^ = bolus dose is moderate, while infusion dose is low*

Arikci, D., Holze, F., Mueller, L., Vizeli, P., Rudin, D., Luethi, D., Hysek, C. M., & Liechti, M. E. (2025). Absolute Oral Bioavailability and Bioequivalence of LSD Base and Tartrate in a Double-Blind, Placebo-Controlled, Crossover Study. *Clinical Pharmacology & Therapeutics*, *n/a*(n/a). https://doi.org/10.1002/cpt.3726

Becker, A. M., Holze, F., Grandinetti, T., Klaiber, A., Toedtli, V. E., Kolaczynska, K. E., Duthaler, U., Varghese, N., Eckert, A., Grünblatt, E., & Liechti, M. E. (2022). Acute effects of psilocybin after escitalopram or placebo pretreatment in a randomized, double-blind, placebo-controlled, crossover study in healthy subjects. *Clinical Pharmacology & Therapeutics*, *111*(4), 886–895. https://doi.org/10.1002/cpt.2487

Becker, A. M., Humbert‐Droz, M., Mueller, L., Jelušić, A., Tolev, A., Straumann, I., Avedisian, I., Erne, L., Thomann, J., Luethi, D., Grünblatt, E., Meyer zu Schwabedissen, H., & Liechti, M. E. (2025). Acute Effects and Pharmacokinetics of LSD after Paroxetine or Placebo Pre‐Administration in a Randomized, Double‐Blind, Cross‐Over Phase I Trial. *Clinical Pharmacology and Therapeutics*, *117*(6), 1784–1792. https://doi.org/10.1002/cpt.3618

Becker, A. M., Klaiber, A., Holze, F., Istampoulouoglou, I., Duthaler, U., Varghese, N., Eckert, A., & Liechti, M. E. (2022). Ketanserin reverses the acute response to LSD in a randomized, double-blind, placebo-controlled, crossover study in healthy subjects. *The International Journal of Neuropsychopharmacology*. https://doi.org/10.1093/ijnp/pyac075

Erne, L., Vogt, S. B., Müller, L., Nuraj, A., Becker, A., Klaiber, A., Zuparic, M., Varghese, N., Eckert, A., Rudin, D., Luethi, D., & Liechti, M. E. (2024). Acute dose-dependent effects and self-guided titration of continuous N,N-dimethyltryptamine infusions in a double-blind placebo-controlled study in healthy participants. *Neuropsychopharmacology*, 1–9. https://doi.org/10.1038/s41386-024-02041-8

Holze, F., Ley, L., Müller, F., Becker, A. M., Straumann, I., Vizeli, P., Kuehne, S. S., Roder, M. A., Duthaler, U., Kolaczynska, K. E., Varghese, N., Eckert, A., & Liechti, M. E. (2022). Direct comparison of the acute effects of lysergic acid diethylamide and psilocybin in a double-blind placebo-controlled study in healthy subjects. *Neuropsychopharmacology*, *47*(6), Article 6. https://doi.org/10.1038/s41386-022-01297-2

Holze, F., Vizeli, P., Ley, L., Müller, F., Dolder, P., Stocker, M., Duthaler, U., Varghese, N., Eckert, A., Borgwardt, S., & Liechti, M. E. (2021). Acute dose-dependent effects of lysergic acid diethylamide in a double-blind placebo-controlled study in healthy subjects. *Neuropsychopharmacology*, *46*(3), 537–544. https://doi.org/10.1038/s41386-020-00883-6

Holze, F., Vizeli, P., Müller, F., Ley, L., Duerig, R., Varghese, N., Eckert, A., Borgwardt, S., & Liechti, M. E. (2020). Distinct acute effects of LSD, MDMA, and D-amphetamine in healthy subjects. *Neuropsychopharmacology*, *45*(3), 462–471. https://doi.org/10.1038/s41386-019-0569-3

Klaiber, A., Schmid, Y., Becker, A. M., Straumann, I., Erne, L., Jelusic, A., Thomann, J., Luethi, D., & Liechti, M. E. (2024). Acute dose-dependent effects of mescaline in a double-blind placebo-controlled study in healthy subjects. *Translational Psychiatry*, *14*(1), 1–8. https://doi.org/10.1038/s41398-024-03116-2

Ley, L., Holze, F., Arikci, D., Becker, A. M., Straumann, I., Klaiber, A., Coviello, F., Dierbach, S., Thomann, J., & Duthaler, U. (2023). Comparative acute effects of mescaline, lysergic acid diethylamide, and psilocybin in a randomized, double-blind, placebo-controlled cross-over study in healthy participants. *Neuropsychopharmacology*, 1–9.

Liechti, M. E., Dolder, P. C., & Schmid, Y. (2017). Alterations of consciousness and mystical-type experiences after acute LSD in humans. *Psychopharmacology*, *234*(9), 1499–1510. https://doi.org/10.1007/s00213-016-4453-0

Schmid, Y., Enzler, F., Gasser, P., Grouzmann, E., Preller, K. H., Vollenweider, F. X., Brenneisen, R., Müller, F., Borgwardt, S., & Liechti, M. E. (2015). Acute Effects of Lysergic Acid Diethylamide in Healthy Subjects. *Biological Psychiatry*, *78*(8), 544–553. https://doi.org/10.1016/j.biopsych.2014.11.015

Straumann, I., Ley, L., Holze, F., Becker, A. M., Klaiber, A., Wey, K., Duthaler, U., Varghese, N., Eckert, A., & Liechti, M. E. (2023). Acute effects of MDMA and LSD co-administration in a double-blind placebo-controlled study in healthy participants. *Neuropsychopharmacology*, *48*(13), 1840–1848. https://doi.org/10.1038/s41386-023-01609-0

Vogt, S. B., Ley, L., Erne, L., Straumann, I., Becker, A. M., Klaiber, A., Holze, F., Vandersmissen, A., Mueller, L., & Duthaler, U. (2023). Acute effects of intravenous DMT in a randomized placebo-controlled study in healthy participants. *Translational Psychiatry*, *13*(1), 172.
