# Supplementary material for: The 3D-ASCr scale: A revalidation of the core dimensions of the Altered States of Consciousness Rating Scale 5D(11)-ASC for psychedelic research
Source: J Psychopharmacol. 2025 Dec 26;40(5):850–62. doi: 10.1177/02698811251397328 (PMC13310268; doi:10.1177/02698811251397328)
Supplement: sj-docx-3-jop-10.1177_02698811251397328 – Supplemental material for The 3D-ASCr scale: A revalidation of the core dimensions of the Altered States of Consciousness Rating Scale 5D(11)-ASC for psychedelic research [file sj-docx-3-jop-10.1177_02698811251397328.docx]

5D-ASC

Five-Dimensional Altered States of Consciousness Scale

German and English Versions

Pages 2–10: German version of the scale, as formally established by Adolf Dittrich, Daniel Lamparter, and Maja Maurer (Dittrich, 1998; Dittrich et al., 2006, 2010). Two items have been slightly revised by Kurt Stocker to improve conceptual clarity (Item 59) and update the language for contemporary use (Item 87).

Pages 11–19: English version of the scale, based on the translation by Felix Hasler and Rael Cahn, with subsequent linguistic refinements led by Kurt Stocker.

Forty-two selected items from the 94-item 5D-ASC form the eleven subscales identified by Studerus and colleagues (2010), from which the three higher-order dimensions of the 3D-ASCr are computed.

The two minor German revisions are as follows: Item 59 was slightly rephrased to avoid semantic ambiguity (“Ich fühlte mich, als wäre ich in einer Art Zustand, den ich vor dem Einschlafen erlebe” instead of “Ich fühlte mich wie vor dem Einschlafen”), and Item 87 was complemented with a contemporary synonym to aid comprehensibility (“Alles um mich herum schien beseelt (belebt)” instead of “Alles um mich herum schien beseelt”).

For the English translation, we used the version by Hasler and Cahn (peer translation) as a basis. All 94 items were carefully reviewed by a native English speaker with professional experience as an English/German translator and the first author (native German speaker with high command of English). The refinements were purely linguistic (closer alignment with the German original and more natural English phrasing) and did not affect the content or psychometric meaning of the items. Thus, data previously collected with the Hasler and Cahn version and data collected with the present version remain directly comparable and can be pooled. The same applies to the other existing English translation of the 5D-ASC by Dittrich, Lamparter, and Maurer, which is sometimes also used, and is highly similar to the version of Hasler and Cahn.

A scoring key for the 3D-ASCr is provided in a separate document.

Dittrich, A. (1998). The standardized psychometric assessment of altered states of consciousness (ASCs) in humans. *Pharmacopsychiatry*, *31*(S 2), 80–84. https://doi.org/10.1055/s-2007-979351

Dittrich, A., Lamparter, D., & Maurer, M. (2006). *5D-ABZ: Fragebogen zur Erfassung Aussergewöhnlicher Bewusstseinszustände: Eine kurze Einführung*. PSIN Plus.

Dittrich, A., Lamparter, D., & Maurer, M. (2010). *5D-ASC: Questionnaire for the assessment of altered states of consciousness. A short introduction*. PSIN Plus.

Studerus, E., Gamma, A., & Vollenweider, F. X. (2010). Psychometric evaluation of the altered states of consciousness rating scale (OAV). *PLoS One*, *5*(8), e12412. https://doi.org/10.1371/journal.pone.0012412

| **5D-ASC** | **T: Xh** |
| --- | --- |

Sie finden auf den folgenden Seiten eine Reihe von Aussagen, unter denen es jeweils eine Linie hat mit den Endpolen „**NEIN**, nicht mehr als gewöhnlich“ und „**JA**, sehr viel mehr als gewöhnlich“. Diese Linie ist eine Skala (0–100%), welche die Abweichung von Ihrem gewöhnlichen und alltäglichen Wachzustand erfassen soll.

**Bitte beurteilen Sie nun, inwieweit Ihr Zustand und Ihre Gefühle vom Beginn bis zum Ende der Wirkung der Substanz im Vergleich zu Ihrem gewöhnlichen und alltäglichen Erleben abgewichen sind.**

Markieren Sie einfach auf der Linie die entsprechende Stelle mit einem **senkrechten Strich**. Sie dürfen alle Zwischenstufen benützen. Wenn eine Aussage zu 0% oder zu 100% zutrifft, können Sie auch das entsprechende Ende der Linie durch Einkreisen markieren.

Beachten Sie bitte, dass Ihr **gewöhnlicher und alltäglicher Wachzustand** dem linken Ende der Linie (0% auf der Skala) entspricht.

**Bitte beurteilen Sie nun die Abweichung Ihres Zustandes und Ihrer Gefühle gegenüber Ihrem gewöhnlichen und alltäglichen Erleben vom Beginn bis zum Ende der Wirkung der Substanz.**

| 1 | **Ich fühlte mich wie in einer wunderbaren anderen Welt.** | | |
| --- | --- | --- | --- |
|  | **NEIN**, nicht mehr als gewöhnlich |  | **JA**, sehr viel mehr als gewöhnlich |
| 2 | **Meine Gedanken und Handlungen waren verlangsamt.** | | |
|  | **NEIN**, nicht mehr als gewöhnlich |  | **JA**, sehr viel mehr als gewöhnlich |
| 3 | **Körperliche Empfindungen waren sehr lustvoll.** | | |
|  | **NEIN**, nicht mehr als gewöhnlich |  | **JA**, sehr viel mehr als gewöhnlich |
| 4 | **Ich hörte einzelne Worte, ohne dass ich wusste, woher sie kamen.** | | |
|  | **NEIN**, nicht mehr als gewöhnlich |  | **JA**, sehr viel mehr als gewöhnlich |
| 5 | **Ich hörte Töne und Klänge, ohne dass ich wusste, woher sie kamen.** | | |
|  | **NEIN**, nicht mehr als gewöhnlich |  | **JA**, sehr viel mehr als gewöhnlich |
| 6 | **Ich fühlte mich dunklen Mächten ausgeliefert.** | | |
|  | **NEIN**, nicht mehr als gewöhnlich |  | **JA**, sehr viel mehr als gewöhnlich |
| 7 | **Ich sah Dinge, von denen ich wusste, dass sie nicht wirklich waren.** | | |
|  | **NEIN**, nicht mehr als gewöhnlich |  | **JA**, sehr viel mehr als gewöhnlich |
| 8 | **Ich fühlte mich wie eine Marionette.** | | |
|  | **NEIN**, nicht mehr als gewöhnlich |  | **JA**, sehr viel mehr als gewöhnlich |

| 9 | **Ich hatte das Gefühl, mit einer höheren Macht verbunden zu sein.** | | |
| --- | --- | --- | --- |
|  | **NEIN**, nicht mehr als gewöhnlich |  | **JA**, sehr viel mehr als gewöhnlich |
| 10 | **Ich fühlte mich schläfrig.** | | |
|  | **NEIN**, nicht mehr als gewöhnlich |  | **JA**, sehr viel mehr als gewöhnlich |
| 11 | **Mir ging eine Melodie im Kopf herum.** | | |
|  | **NEIN**, nicht mehr als gewöhnlich |  | **JA**, sehr viel mehr als gewöhnlich |
| 12 | **Ich empfand grenzenlose Freude.** | | |
|  | **NEIN**, nicht mehr als gewöhnlich |  | **JA**, sehr viel mehr als gewöhnlich |
| 13 | **Sinnlose Geräusche klangen wie richtige Wörter oder Sätze.** | | |
|  | **NEIN**, nicht mehr als gewöhnlich |  | **JA**, sehr viel mehr als gewöhnlich |
| 14 | **Ich sah in völliger Dunkelheit oder mit geschlossenen Augen regelmässige Muster.** | | |
|  | **NEIN**, nicht mehr als gewöhnlich |  | **JA**, sehr viel mehr als gewöhnlich |
| 15 | **Ich fühlte mich wie betrunken.** | | |
|  | **NEIN**, nicht mehr als gewöhnlich |  | **JA**, sehr viel mehr als gewöhnlich |
| 16 | **Ich dachte, ich würde mich nachhaltig auf wunderbare Weise verändern.** | | |
|  | **NEIN**, nicht mehr als gewöhnlich |  | **JA**, sehr viel mehr als gewöhnlich |
| 17 | **Ich fühlte mich am Rande der Bewusstlosigkeit.** | | |
|  | **NEIN**, nicht mehr als gewöhnlich |  | **JA**, sehr viel mehr als gewöhnlich |
| 18 | **Alle Dinge schienen sich zu einem Ganzen zu vereinen.** | | |
|  | **NEIN**, nicht mehr als gewöhnlich |  | **JA**, sehr viel mehr als gewöhnlich |
| 19 | **Ich hörte meine Gedanken, als ob ich laut gesprochen hätte.** | | |
|  | **NEIN**, nicht mehr als gewöhnlich |  | **JA**, sehr viel mehr als gewöhnlich |
| 20 | **Töne schienen das, was ich sah, zu beeinflussen.** | | |
|  | **NEIN**, nicht mehr als gewöhnlich |  | **JA**, sehr viel mehr als gewöhnlich |

| 21 | **Ich fühlte mich gequält.** | | |
| --- | --- | --- | --- |
|  | **NEIN**, nicht mehr als gewöhnlich |  | **JA**, sehr viel mehr als gewöhnlich |
| 22 | **Ich sah in völliger Dunkelheit oder mit geschlossenen Augen Farben vor mir.** | | |
|  | **NEIN**, nicht mehr als gewöhnlich |  | **JA**, sehr viel mehr als gewöhnlich |
| 23 | **Formen schienen sich durch Töne oder Geräusche zu verändern.** | | |
|  | **NEIN**, nicht mehr als gewöhnlich |  | **JA**, sehr viel mehr als gewöhnlich |
| 24 | **Ich erlebte alles unklar, wie in einer Art Nebel.** | | |
|  | **NEIN**, nicht mehr als gewöhnlich |  | **JA**, sehr viel mehr als gewöhnlich |
| 25 | **Eine Stimme kommentierte alles, was ich dachte, obwohl niemand da war.** | | |
|  | **NEIN**, nicht mehr als gewöhnlich |  | **JA**, sehr viel mehr als gewöhnlich |
| 26 | **Ich fühlte mich körperlos.** | | |
|  | **NEIN**, nicht mehr als gewöhnlich |  | **JA**, sehr viel mehr als gewöhnlich |
| 27 | **Ich war unfähig, auch nur die kleinste Entscheidung zu treffen.** | | |
|  | **NEIN**, nicht mehr als gewöhnlich |  | **JA**, sehr viel mehr als gewöhnlich |
| 28 | **Manche Nebensächlichkeiten hatten eine besondere Bedeutung.** | | |
|  | **NEIN**, nicht mehr als gewöhnlich |  | **JA**, sehr viel mehr als gewöhnlich |
| 29 | **Ich fühlte mich dösig.** | | |
|  | **NEIN**, nicht mehr als gewöhnlich |  | **JA**, sehr viel mehr als gewöhnlich |
| 30 | **Ich hörte ganze Sätze, ohne dass ich wusste, woher sie kamen.** | | |
|  | **NEIN**, nicht mehr als gewöhnlich |  | **JA**, sehr viel mehr als gewöhnlich |
| 31 | **Dinge in meiner Umgebung hatten für mich eine neue, fremdartige Bedeutung.** | | |
|  | **NEIN**, nicht mehr als gewöhnlich |  | **JA**, sehr viel mehr als gewöhnlich |
| 32 | **Ich hatte Angst, aus meinem Zustand nicht mehr herauszukommen.** | | |
|  | **NEIN**, nicht mehr als gewöhnlich |  | **JA**, sehr viel mehr als gewöhnlich |

| 33 | **Ich sah in völliger Dunkelheit oder mit geschlossenen Augen Helligkeit oder Lichtblitze.** | | |
| --- | --- | --- | --- |
|  | **NEIN**, nicht mehr als gewöhnlich |  | **JA**, sehr viel mehr als gewöhnlich |
| 34 | **Ich fühlte mich eins mit meiner Umgebung.** | | |
|  | **NEIN**, nicht mehr als gewöhnlich |  | **JA**, sehr viel mehr als gewöhnlich |
| 35 | **Sorgen und Ängste des Alltags kamen mir belanglos vor.** | | |
|  | **NEIN**, nicht mehr als gewöhnlich |  | **JA**, sehr viel mehr als gewöhnlich |
| 36 | **Wie im Traum waren Raum- und Zeitgefühl verändert.** | | |
|  | **NEIN**, nicht mehr als gewöhnlich |  | **JA**, sehr viel mehr als gewöhnlich |
| 37 | **Meine Wahrnehmung war getrübt.** | | |
|  | **NEIN**, nicht mehr als gewöhnlich |  | **JA**, sehr viel mehr als gewöhnlich |
| 38 | **Es fiel mir schwer, Wichtiges von Unwichtigem zu unterscheiden.** | | |
|  | **NEIN**, nicht mehr als gewöhnlich |  | **JA**, sehr viel mehr als gewöhnlich |
| 39 | **Ich sah in völliger Dunkelheit oder mit geschlossenen Augen ganze Szenen.** | | |
|  | **NEIN**, nicht mehr als gewöhnlich |  | **JA**, sehr viel mehr als gewöhnlich |
| 40 | **Ich fühlte aussergewöhnliche Kraft in mir.** | | |
|  | **NEIN**, nicht mehr als gewöhnlich |  | **JA**, sehr viel mehr als gewöhnlich |
| 41 | **Ich verspürte einen Hauch von Ewigkeit.** | | |
|  | **NEIN**, nicht mehr als gewöhnlich |  | **JA**, sehr viel mehr als gewöhnlich |
| 42 | **Gegensätze und Widersprüche schienen sich aufzulösen.** | | |
|  | **NEIN**, nicht mehr als gewöhnlich |  | **JA**, sehr viel mehr als gewöhnlich |
| 43 | **Ich hatte Angst, ohne genau sagen zu können weshalb.** | | |
|  | **NEIN**, nicht mehr als gewöhnlich |  | **JA**, sehr viel mehr als gewöhnlich |
| 44 | **Ich erlebte alles beängstigend verzerrt.** | | |
|  | **NEIN**, nicht mehr als gewöhnlich |  | **JA**, sehr viel mehr als gewöhnlich |

| 45 | **Die Welt schien mir jenseits von Gut und Böse.** | | |
| --- | --- | --- | --- |
|  | **NEIN**, nicht mehr als gewöhnlich |  | **JA**, sehr viel mehr als gewöhnlich |
| 46 | **Meine Umgebung kam mir fremd und unheimlich vor.** | | |
|  | **NEIN**, nicht mehr als gewöhnlich |  | **JA**, sehr viel mehr als gewöhnlich |
| 47 | **Ich fühlte mich wie gelähmt.** | | |
|  | **NEIN**, nicht mehr als gewöhnlich |  | **JA**, sehr viel mehr als gewöhnlich |
| 48 | **Ich hörte Musik, ohne dass ich wusste, woher sie kam.** | | |
|  | **NEIN**, nicht mehr als gewöhnlich |  | **JA**, sehr viel mehr als gewöhnlich |
| 49 | **Ich hörte ganz leise etwas, von dem ich nicht sagen konnte, was es war.** | | |
|  | **NEIN**, nicht mehr als gewöhnlich |  | **JA**, sehr viel mehr als gewöhnlich |
| 50 | **Ich kam mir besonders tiefgründig vor.** | | |
|  | **NEIN**, nicht mehr als gewöhnlich |  | **JA**, sehr viel mehr als gewöhnlich |
| 51 | **Ich fühlte mich benommen.** | | |
|  | **NEIN**, nicht mehr als gewöhnlich |  | **JA**, sehr viel mehr als gewöhnlich |
| 52 | **Vergangenheit, Gegenwart und Zukunft erlebte ich als eine Einheit.** | | |
|  | **NEIN**, nicht mehr als gewöhnlich |  | **JA**, sehr viel mehr als gewöhnlich |
| 53 | **Ich hatte das Gefühl einer unerträglichen Leere.** | | |
|  | **NEIN**, nicht mehr als gewöhnlich |  | **JA**, sehr viel mehr als gewöhnlich |
| 54 | **Gegenstände in meiner Umgebung sprachen mich gefühlsmässig viel stärker an.** | | |
|  | **NEIN**, nicht mehr als gewöhnlich |  | **JA**, sehr viel mehr als gewöhnlich |
| 55 | **Aus einem anfänglich diffusen Geräusch, von dem ich nicht wusste, ob es wirklich war, entwickelten sich klare Töne und Klänge.** | | |
|  | **NEIN**, nicht mehr als gewöhnlich |  | **JA**, sehr viel mehr als gewöhnlich |
| 56 | **Ich fühlte mich bedroht.** | | |
|  | **NEIN**, nicht mehr als gewöhnlich |  | **JA**, sehr viel mehr als gewöhnlich |

| 57 | **Vieles erschien mir von atemberaubender Schönheit.** | | |
| --- | --- | --- | --- |
|  | **NEIN**, nicht mehr als gewöhnlich |  | **JA**, sehr viel mehr als gewöhnlich |
| 58 | **Mir kamen Dinge in den Sinn, die ich schon lange vergessen glaubte.** | | |
|  | **NEIN**, nicht mehr als gewöhnlich |  | **JA**, sehr viel mehr als gewöhnlich |
| 59 | **Ich fühlte mich, als wäre ich in einer Art Zustand, den ich vor dem Einschlafen erlebe.** | | |
|  | **NEIN**, nicht mehr als gewöhnlich |  | **JA**, sehr viel mehr als gewöhnlich |
| 60 | **Mein Körper erschien mir gefühllos, leblos, fremd.** | | |
|  | **NEIN**, nicht mehr als gewöhnlich |  | **JA**, sehr viel mehr als gewöhnlich |
| 61 | **Ich war in einer Art Halbschlaf.** | | |
|  | **NEIN**, nicht mehr als gewöhnlich |  | **JA**, sehr viel mehr als gewöhnlich |
| 62 | **Ich hatte das Gefühl, ausserhalb meines Körpers zu sein.** | | |
|  | **NEIN**, nicht mehr als gewöhnlich |  | **JA**, sehr viel mehr als gewöhnlich |
| 63 | **Ich fühlte mich, als ob ich schweben würde.** | | |
|  | **NEIN**, nicht mehr als gewöhnlich |  | **JA**, sehr viel mehr als gewöhnlich |
| 64 | **Ich fühlte mich isoliert von allem und jedem.** | | |
|  | **NEIN**, nicht mehr als gewöhnlich |  | **JA**, sehr viel mehr als gewöhnlich |
| 65 | **Ich hörte Stimmen, die nicht wie üblich aus der Umgebung kamen.** | | |
|  | **NEIN**, nicht mehr als gewöhnlich |  | **JA**, sehr viel mehr als gewöhnlich |
| 66 | **Ich hörte ein Summen, Rauschen, Brummen oder Ähnliches, ohne dass ich die Ursache dafür erkennen konnte.** | | |
|  | **NEIN**, nicht mehr als gewöhnlich |  | **JA**, sehr viel mehr als gewöhnlich |
| 67 | **Meine Gedanken rissen immer wieder ab, ich konnte nichts richtig zu Ende denken.** | | |
|  | **NEIN**, nicht mehr als gewöhnlich |  | **JA**, sehr viel mehr als gewöhnlich |
| 68 | **Ich glaubte, ich würde gleich einschlafen.** | | |
|  | **NEIN**, nicht mehr als gewöhnlich |  | **JA**, sehr viel mehr als gewöhnlich |

| 69 | **Ich gewann Einsichten in Zusammenhänge, die mir vorher rätselhaft waren.** | | |
| --- | --- | --- | --- |
|  | **NEIN**, nicht mehr als gewöhnlich |  | **JA**, sehr viel mehr als gewöhnlich |
| 70 | **Vieles kam mir unglaublich witzig vor.** | | |
|  | **NEIN**, nicht mehr als gewöhnlich |  | **JA**, sehr viel mehr als gewöhnlich |
| 71 | **Die Grenze zwischen mir und meiner Umgebung schien sich zu verwischen.** | | |
|  | **NEIN**, nicht mehr als gewöhnlich |  | **JA**, sehr viel mehr als gewöhnlich |
| 72 | **Ich konnte Bilder aus der Erinnerung oder aus der Fantasie überaus deutlich sehen.** | | |
|  | **NEIN**, nicht mehr als gewöhnlich |  | **JA**, sehr viel mehr als gewöhnlich |
| 73 | **Ich fühlte mich vollkommen frei und losgelöst von allen Verpflichtungen.** | | |
|  | **NEIN**, nicht mehr als gewöhnlich |  | **JA**, sehr viel mehr als gewöhnlich |
| 74 | **Ich hörte diffuse Geräusche, ohne dass ich wusste woher sie kamen.** | | |
|  | **NEIN**, nicht mehr als gewöhnlich |  | **JA**, sehr viel mehr als gewöhnlich |
| 75 | **Farben schienen sich durch Töne oder Geräusche zu verändern.** | | |
|  | **NEIN**, nicht mehr als gewöhnlich |  | **JA**, sehr viel mehr als gewöhnlich |
| 76 | **Töne und Geräusche klangen leiser als sonst.** | | |
|  | **NEIN**, nicht mehr als gewöhnlich |  | **JA**, sehr viel mehr als gewöhnlich |
| 77 | **Ich hatte besonders originelle Einfälle.** | | |
|  | **NEIN**, nicht mehr als gewöhnlich |  | **JA**, sehr viel mehr als gewöhnlich |
| 78 | **Ich hatte das Gefühl, keinen eigenen Willen mehr zu haben.** | | |
|  | **NEIN**, nicht mehr als gewöhnlich |  | **JA**, sehr viel mehr als gewöhnlich |
| 79 | **Ich hatte Angst, die Kontrolle über mich zu verlieren.** | | |
|  | **NEIN**, nicht mehr als gewöhnlich |  | **JA**, sehr viel mehr als gewöhnlich |
| 80 | **Ich verharrte längere Zeit wie erstarrt in einer ganz unnatürlichen Haltung.** | | |
|  | **NEIN**, nicht mehr als gewöhnlich |  | **JA**, sehr viel mehr als gewöhnlich |

| 81 | **Ich empfand ein Gefühl der Ehrfurcht.** | | |
| --- | --- | --- | --- |
|  | **NEIN**, nicht mehr als gewöhnlich |  | **JA**, sehr viel mehr als gewöhnlich |
| 82 | **Meine Fantasie war äusserst lebhaft.** | | |
|  | **NEIN**, nicht mehr als gewöhnlich |  | **JA**, sehr viel mehr als gewöhnlich |
| 83 | **Die Dinge um mich herum erschienen mir kleiner oder grösser.** | | |
|  | **NEIN**, nicht mehr als gewöhnlich |  | **JA**, sehr viel mehr als gewöhnlich |
| 84 | **Ich fühlte mich erschöpft.** | | |
|  | **NEIN**, nicht mehr als gewöhnlich |  | **JA**, sehr viel mehr als gewöhnlich |
| 85 | **Die Zeit verging quälend langsam.** | | |
|  | **NEIN**, nicht mehr als gewöhnlich |  | **JA**, sehr viel mehr als gewöhnlich |
| 86 | **Ich empfand tiefen Frieden in mir.** | | |
|  | **NEIN**, nicht mehr als gewöhnlich |  | **JA**, sehr viel mehr als gewöhnlich |
| 87 | **Alles um mich herum schien beseelt (belebt).** | | |
|  | **NEIN**, nicht mehr als gewöhnlich |  | **JA**, sehr viel mehr als gewöhnlich |
| 88 | **Alles ging so schnell, dass ich mich nicht mehr zurecht fand.** | | |
|  | **NEIN**, nicht mehr als gewöhnlich |  | **JA**, sehr viel mehr als gewöhnlich |
| 89 | **Ich hatte das Gefühl, es würde Schreckliches geschehen.** | | |
|  | **NEIN**, nicht mehr als gewöhnlich |  | **JA**, sehr viel mehr als gewöhnlich |
| 90 | **Ich konnte mich an bestimmte Ereignisse überdeutlich erinnern.** | | |
|  | **NEIN**, nicht mehr als gewöhnlich |  | **JA**, sehr viel mehr als gewöhnlich |
| 91 | **Ich empfand eine allumfassende Liebe.** | | |
|  | **NEIN**, nicht mehr als gewöhnlich |  | **JA**, sehr viel mehr als gewöhnlich |
| 92 | **Es waren Geräusche im Raum, von denen ich kaum glaube, dass sie existierten.** | | |
|  | **NEIN**, nicht mehr als gewöhnlich |  | **JA**, sehr viel mehr als gewöhnlich |

| 93 | **Ich hörte ein Ticken, Klopfen, Rattern oder Ähnliches, ohne dass ich die Ursache dafür erkennen konnte.** | | |
| --- | --- | --- | --- |
|  | **NEIN**, nicht mehr als gewöhnlich |  | **JA**, sehr viel mehr als gewöhnlich |
| 94 | **Mein Erleben hatte religiösen Charakter.** | | |
|  | **NEIN**, nicht mehr als gewöhnlich |  | **JA**, sehr viel mehr als gewöhnlich |

| **5D-ASC** | **T: Xh** |
| --- | --- |

On the following pages, you will find a series of statements and, below them, a line with the endpoints “**NO**, no more than usual” and “**YES**, much more than usual.” The line is a scale (0–100%) designed to measure the deviation from your usual, everyday waking state.

**Please now assess the extent to which your state and feelings deviated from your usual, everyday experience from the beginning to the end of the effects of the substance.**

Simply mark the appropriate point on the scale with a **vertical stroke**. You may use any intermediate value. If a statement applies 0% or 100%, you may also indicate this by circling the appropriate end of the line.

Please note that your **usual, everyday waking state** corresponds to the line on the far left (0% on the scale).

**Please now assess the deviation of your state and feelings from your usual, everyday experience from the beginning to the end of the effects of the substance.**

| 1 | **I felt as if I were in a wonderful, other world.** | | |
| --- | --- | --- | --- |
|  | **NO**, no more than usual |  | **YES**, much more than usual |
| 2 | **My thoughts and actions were slowed down.** | | |
|  | **NO,** no more than usual |  | **YES**, much more than usual |
| 3 | **Physical sensations were very pleasurable.** | | |
|  | **NO,** no more than usual |  | **YES**, much more than usual |
| 4 | **I heard single words without knowing where they came from.** | | |
|  | **NO**, no more than usual |  | **YES**, much more than usual |
| 5 | **I heard tones and sounds without knowing where they came from.** | | |
|  | **NO**, no more than usual |  | **YES**, much more than usual |
| 6 | **I felt at the mercy of dark forces.** | | |
|  | **NO**, no more than usual |  | **YES**, much more than usual |
| 7 | **I saw things I knew were not real.** | | |
|  | **NO**, no more than usual |  | **YES**, much more than usual |
| 8 | **I felt like a puppet or marionette.** | | |
|  | **NO**, no more than usual |  | **YES**, much more than usual |

| 9 | **I had the feeling of being connected to a higher power.** | | |
| --- | --- | --- | --- |
|  | **NO**, no more than usual |  | **YES**, much more than usual |
| 10 | **I felt sleepy.** | | |
|  | **NO**, no more than usual |  | **YES**, much more than usual |
| 11 | **A melody kept going through my head.** | | |
|  | **NO**, no more than usual |  | **YES**, much more than usual |
| 12 | **I experienced boundless joy.** | | |
|  | **NO**, no more than usual |  | **YES**, much more than usual |
| 13 | **Meaningless noises sounded like real words or phrases.** | | |
|  | **NO**, no more than usual |  | **YES**, much more than usual |
| 14 | **I saw regular patterns in complete darkness or with closed eyes.** | | |
|  | **NO**, no more than usual |  | **YES**, much more than usual |
| 15 | **I felt as if I were drunk.** | | |
|  | **NO**, no more than usual |  | **YES**, much more than usual |
| 16 | **I believed I was undergoing a lasting and miraculous transformation.** | | |
|  | **NO**, no more than usual |  | **YES**, much more than usual |
| 17 | **I felt that I was on the verge of unconsciousness.** | | |
|  | **NO**, no more than usual |  | **YES**, much more than usual |
| 18 | **Everything seemed to unify into oneness.** | | |
|  | **NO**, no more than usual |  | **YES**, much more than usual |
| 19 | **I heard my thoughts as if I had spoken them out loud.** | | |
|  | **NO**, no more than usual |  | **YES**, much more than usual |
| 20 | **Sounds seemed to influence what I saw.** | | |
|  | **NO**, no more than usual |  | **YES**, much more than usual |

| 21 | **I felt tormented.** | | |
| --- | --- | --- | --- |
|  | **NO**, no more than usual |  | **YES**, much more than usual |
| 22 | **I saw colors before me in total darkness or with closed eyes.** | | |
|  | **NO**, no more than usual |  | **YES**, much more than usual |
| 23 | **The shapes of things seemed to be changed by sounds or noises.** | | |
|  | **NO**, no more than usual |  | **YES**, much more than usual |
| 24 | **I perceived everything as blurry, as if through a kind of fog.** | | |
|  | **NO**, no more than usual |  | **YES**, much more than usual |
| 25 | **A voice commented on everything I thought, even though no one was there.** | | |
|  | **NO**, no more than usual |  | **YES**, much more than usual |
| 26 | **I felt bodiless.** | | |
|  | **NO, no more than usual** |  | **YES**, much more than usual |
| 27 | **I was unable to make even the smallest decision.** | | |
|  | **NO**, no more than usual |  | **YES**, much more than usual |
| 28 | **Some everyday things gained a special meaning.** | | |
|  | **NO**, no more than usual |  | **YES**, much more than usual |
| 29 | **I felt drowsy.** | | |
|  | **NO**, no more than usual |  | **YES**, much more than usual |
| 30 | **I heard entire sentences without knowing where they came from.** | | |
|  | **NO**, no more than usual |  | **YES**, much more than usual |
| 31 | **Things around me had a new, unfamiliar meaning for me.** | | |
|  | **NO**, no more than usual |  | **YES**, much more than usual |
| 32 | **I was afraid I wouldn’t be able to get out of the state I was in.** | | |
|  | **NO**, no more than usual |  | **YES**, much more than usual |

| 33 | **I saw brightness or flashes of light in total darkness or with closed eyes.** | | |
| --- | --- | --- | --- |
|  | **NO**, no more than usual |  | **YES**, much more than usual |
| 34 | **I felt at one with my surroundings.** | | |
|  | **NO**, no more than usual |  | **YES**, much more than usual |
| 35 | **Everyday worries and fears felt trivial to me.** | | |
|  | **NO**, no more than usual |  | **YES**, much more than usual |
| 36 | **My sense of time and space was altered, as if in a dream.** | | |
|  | **NO**, no more than usual |  | **YES**, much more than usual |
| 37 | **My perception was clouded.** | | |
|  | **NO**, no more than usual |  | **YES**, much more than usual |
| 38 | **I had difficulties in distinguishing important from unimportant things.** | | |
|  | **NO**, no more than usual |  | **YES**, much more than usual |
| 39 | **I saw entire scenes in total darkness or with my eyes closed.** | | |
|  | **NO**, no more than usual |  | **YES**, much more than usual |
| 40 | **I felt an extraordinary power within myself.** | | |
|  | **NO**, no more than usual |  | **YES**, much more than usual |
| 41 | **I sensed a touch of eternity.** | | |
|  | **NO**, no more than usual |  | **YES**, much more than usual |
| 42 | **Opposites and contradictions seemed to dissolve.** | | |
|  | **NO**, no more than usual |  | **YES**, much more than usual |
| 43 | **I was afraid without being able to say exactly why.** | | |
|  | **NO**, no more than usual |  | **YES**, much more than usual |
| 44 | **I experienced everything as frighteningly distorted.** | | |
|  | **NO**, no more than usual |  | **YES**, much more than usual |

| 45 | **The world seemed to me to be beyond good and evil.** | | |
| --- | --- | --- | --- |
|  | **NO**, no more than usual |  | **YES**, much more than usual |
| 46 | **I experienced my surroundings as strange and unsettling.** | | |
|  | **NO**, no more than usual |  | **YES**, much more than usual |
| 47 | **I felt as if I were paralyzed.** | | |
|  | **NO**, no more than usual |  | **YES**, much more than usual |
| 48 | **I heard music without knowing where it came from.** | | |
|  | **NO**, no more than usual |  | **YES**, much more than usual |
| 49 | **I heard something very faint that I could not identify.** | | |
|  | **NO**, no more than usual |  | **YES**, much more than usual |
| 50 | **I felt like I was in a particularly profound state.** | | |
|  | **NO**, no more than usual |  | **YES**, much more than usual |
| 51 | **I felt dazed.** | | |
|  | **NO**, no more than usual |  | **YES**, much more than usual |
| 52 | **I experienced past, present and future as one.** | | |
|  | **NO**, no more than usual |  | **YES**, much more than usual |
| 53 | **I had the feeling of an unbearable void.** | | |
|  | **NO**, no more than usual |  | **YES**, much more than usual |
| 54 | **Objects around me engaged me emotionally much more than usual.** | | |
|  | **NO**, no more than usual |  | **YES**, much more than usual |
| 55 | **Clear tones and sounds emerged from an initially indistinct noise, which I was not sure was even real.** | | |
|  | **NO**, no more than usual |  | **YES**, much more than usual |
| 56 | **I felt threatened.** | | |
|  | **NO**, no more than usual |  | **YES**, much more than usual |

| 57 | **Many things appeared breathtakingly beautiful to me.** | | |
| --- | --- | --- | --- |
|  | **NO**, no more than usual |  | **YES**, much more than usual |
| 58 | **Things came to mind that I thought I had long forgotten.** | | |
|  | **NO**, no more than usual |  | **YES**, much more than usual |
| 59 | **I felt as if I were in the kind of state I experience before falling asleep.** | | |
|  | **NO**, no more than usual |  | **YES**, much more than usual |
| 60 | **My body felt numb, lifeless and strange.** | | |
|  | **NO**, no more than usual |  | **YES**, much more than usual |
| 61 | **I felt as if I were in a kind of half-sleep.** | | |
|  | **NO**, no more than usual |  | **YES**, much more than usual |
| 62 | **I had the feeling of being outside of my body.** | | |
|  | **NO**, no more than usual |  | **YES**, much more than usual |
| 63 | **I felt as though I were floating.** | | |
|  | **NO**, no more than usual |  | **YES**, much more than usual |
| 64 | **I felt isolated from everything and everyone.** | | |
|  | **NO**, no more than usual |  | **YES**, much more than usual |
| 65 | **I heard voices that did not come from my surroundings, as they usually do.** | | |
|  | **NO**, no more than usual |  | **YES**, much more than usual |
| 66 | **I heard humming, rushing or buzzing, or similar sounds, without being able to identify the source.** | | |
|  | **NO**, no more than usual |  | **YES**, much more than usual |
| 67 | **My thoughts kept breaking off; I could not think anything through to the end.** | | |
|  | **NO**, no more than usual |  | **YES**, much more than usual |
| 68 | **I thought I was just about to fall asleep.** | | |
|  | **NO**, no more than usual |  | **YES**, much more than usual |

| 69 | **I gained insights into connections that had previously puzzled me.** | | |
| --- | --- | --- | --- |
|  | **NO**, no more than usual |  | **YES**, much more than usual |
| 70 | **Many things seemed incredibly funny to me.** | | |
|  | **NO**, no more than usual |  | **YES**, much more than usual |
| 71 | **The boundaries between myself and my surroundings seemed to blur.** | | |
|  | **NO**, no more than usual |  | **YES**, much more than usual |
| 72 | **I could see images from my memory or imagination with extreme clarity.** | | |
|  | **NO**, no more than usual |  | **YES**, much more than usual |
| 73 | **I felt completely free and detached from all obligations.** | | |
|  | **NO**, no more than usual |  | **YES**, much more than usual |
| 74 | **I heard indistinct noises without knowing where they came from.** | | |
|  | **NO, no more than usual** |  | **YES**, much more than usual |
| 75 | **The colors of things seemed to be changed by sounds or noises.** | | |
|  | **NO**, no more than usual |  | **YES**, much more than usual |
| 76 | **Sounds and noises were fainter than usual.** | | |
|  | **NO**, no more than usual |  | **YES**, much more than usual |
| 77 | **I had particularly original thoughts.** | | |
|  | **NO**, no more than usual |  | **YES**, much more than usual |
| 78 | **I had the feeling that I no longer had a will of my own.** | | |
|  | **NO**, no more than usual |  | **YES**, much more than usual |
| 79 | **I was afraid of losing control of myself.** | | |
|  | **NO**, no more than usual |  | **YES**, much more than usual |
| 80 | **I remained frozen for a long time in a completely unnatural position.** | | |
|  | **NO**, no more than usual |  | **YES**, much more than usual |

| 81 | **I experienced a feeling of awe.** | | |
| --- | --- | --- | --- |
|  | **NO**, no more than usual |  | **YES**, much more than usual |
| 82 | **My imagination was extremely vivid.** | | |
|  | **NO**, no more than usual |  | **YES**, much more than usual |
| 83 | **Things around me seemed smaller or larger.** | | |
|  | **NO**, no more than usual |  | **YES**, much more than usual |
| 84 | **I felt exhausted.** | | |
|  | **NO**, no more than usual |  | **YES**, much more than usual |
| 85 | **Time passed agonizingly slowly.** | | |
|  | **NO**, no more than usual |  | **YES**, much more than usual |
| 86 | **I experienced profound inner peace.** | | |
|  | **NO**, no more than usual |  | **YES**, much more than usual |
| 87 | **Everything around me seemed to be imbued with life.** | | |
|  | **NO**, no more than usual |  | **YES**, much more than usual |
| 88 | **Everything happened so fast that I could not keep up with it all.** | | |
|  | **NO**, no more than usual |  | **YES**, much more than usual |
| 89 | **I had the feeling that something terrible was going to happen.** | | |
|  | **NO**, no more than usual |  | **YES**, much more than usual |
| 90 | **I could remember certain events with striking clarity.** | | |
|  | **NO**, no more than usual |  | **YES**, much more than usual |
| 91 | **I experienced an all-embracing love.** | | |
|  | **NO**, no more than usual |  | **YES**, much more than usual |
| 92 | **There were noises in the room that I could hardly believe were real.** | | |
|  | **NO**, no more than usual |  | **YES**, much more than usual |

| 93 | **I heard ticking, knocking, rattling, or similar sounds, without being able to identify the source.** | | |
| --- | --- | --- | --- |
|  | **NO**, no more than usual |  | **YES**, much more than usual |
| 94 | **My experience was religious in nature.** | | |
|  | **NO**, no more than usual |  | **YES**, much more than usual |
