# Supplementary material for: The 3D-ASCr scale: A revalidation of the core dimensions of the Altered States of Consciousness Rating Scale 5D(11)-ASC for psychedelic research
Source: J Psychopharmacol. 2025 Dec 26;40(5):850–62. doi: 10.1177/02698811251397328 (PMC13310268; doi:10.1177/02698811251397328)
Supplement: sj-docx-4-jop-10.1177_02698811251397328 – Supplemental material for The 3D-ASCr scale: A revalidation of the core dimensions of the Altered States of Consciousness Rating Scale 5D(11)-ASC for psychedelic research [file sj-docx-4-jop-10.1177_02698811251397328.docx]

3D-ASCr Scoring Key

Within the 42-item version of the 3D-ASCr scale

| **Dimensions** | **Subscales** | **Item numbers** |
| --- | --- | --- |
| **Positive Effects (PosE)** | 1. Experience of Unity | 5, 15, 18, 19, 25 |
|  | 2. Spiritual Experience | 2, 37, 42 |
|  | 3. Blissful State | 3, 39, 41 |
|  | 4. Insightfulness | 24, 32, 35 |
|  | 5. Disembodiment | 9, 28, 29 |
|  | 6. Changed Meaning | 11, 12, 26 |
| **Perceptual Effects (PerE)** | 7. Complex Imagery | 17, 33, 38 |
|  | 8. Elementary Imagery | 4, 7, 14 |
|  | 9. Audio-Visual Synesthesiae | 6, 8, 34 |
| **Distressing Effects (DisE)** | 10. Impaired Control and Cognition | 1, 10, 16, 23, 30, 31, 36 |
|  | 11. Anxiety* | 13, 20, 21, 22, 27, 40 |

Within the 42 selected items of the 94-item 5D-ASC questionnaire

| **Dimensions** | **Subscales** | **Item numbers** |
| --- | --- | --- |
| **Positive Effects (PosE)** | 1. Experience of Unity | 18, 34, 41, 42, 52 |
|  | 2. Spiritual Experience | 9, 81, 94 |
|  | 3. Blissful State | 12, 86, 91 |
|  | 4. Insightfulness | 50, 69, 77 |
|  | 5. Disembodiment | 26. 62. 63 |
|  | 6. Changed Meaning | 28, 31, 54 |
| **Perceptual Effects (PerE)** | 7. Complex Imagery | 39, 72, 82 |
|  | 8. Elementary Imagery | 14, 22, 33 |
|  | 9. Audio-Visual Synesthesiae | 20, 23, 76 |
| **Distressing Effects (DisE)** | 10. Impaired Control and Cognition | 8, 27, 38, 47. 64, 67, 78 |
|  | 11. Anxiety* | 32. 43, 44, 46, 56, 89 |

Scoring instruction: First compute the mean scores for each of the eleven subscales. Then compute the scores of the three higher-order dimensions by averaging the subscale means of the respective dimension, as indicated above.

* *Anxiety*: Based on content relatedness and results from our exploratory factor analysis, combining Impaired Control and Cognition and Anxiety into DisE can be reasonable. However, due to consistent floor effects (i.e., low anxiety scores) and model fit issues in the confirmatory factor analysis, the Anxiety subscale may optionally be included in or excluded from DisE, depending on the dataset and research purpose. In case of uncertainty, we recommend reporting DisE both with and without Anxiety. If DisE is reported without Anxiety, the Anxiety subscale should still be reported as a standalone measure, as it still may convey clinically or contextually relevant information.

3D-ASCr Items by Dimension and Subscale

The first item number refers to the 42-item 3D-ASCr version; the number in brackets indicates the corresponding item number from the 94-item 5D-ASC.

Positive Effects (PosE)

| **Factor 1: Experience of Unity** |
| --- |
| 5 [18]. Everything seemed to unify into oneness. |
| 15 [34]. I felt at one with my surroundings. |
| 18 [41]. I sensed a touch of eternity. |
| 19 [42]. Opposites and contradictions seemed to dissolve. |
| 25 [52]. I experienced past, present and future as one. |

| **Factor 2: Spiritual Experience** |
| --- |
| 2 [9]. I had the feeling of being connected to a higher power. |
| 37 [81]. I experienced a feeling of awe. |
| 42 [94]. My experience was religious in nature. |

| **Factor 3: Blissful State** |
| --- |
| 3 [12]. I experienced boundless joy. |
| 39 [86]. I experienced profound inner peace. |
| 41 [91]. I experienced an all-embracing love. |

| **Factor 4: Insightfulness** |
| --- |
| 24 [50]. I felt like I was in a particularly profound state. |
| 32 [69]. I gained insights into connections that had previously puzzled me. |
| 35 [77]. I had particularly original thoughts. |

| **Factor 5: Disembodiment** |
| --- |
| 9 [26]. I felt bodiless. |
| 28 [62]. I had the feeling of being outside of my body. |
| 29 [63]. I felt as though I were floating. |

| **Factor 6: Changed Meaning** |
| --- |
| 11 [28]. Some everyday things gained a special meaning. |
| 12 [31]. Things around me had a new, unfamiliar meaning for me. |
| 26 [54]. Objects around me engaged me emotionally much more than usual. |

Perceptual Effects (PerE)

| **Factor 7: Complex Imagery** |
| --- |
| 17 [39]. I saw entire scenes in total darkness or with my eyes closed. |
| 33 [72]. I could see images from my memory or imagination with extreme clarity. |
| 38 [82]. My imagination was extremely vivid. |

| **Factor 8: Elementary Imagery** |
| --- |
| 4 [14]. I saw regular patterns in complete darkness or with closed eyes. |
| 7 [22]. I saw colors before me in complete darkness or with closed eyes. |
| 14 [33]. I saw brightness or flashes of light in complete darkness or with closed eyes. |

| **Factor 9: Audio-Visual Synesthesiae** |
| --- |
| 6 [20]. Sounds seemed to influence what I saw. |
| 8 [23]. The shapes of things seemed to be changed by sounds or noises. |
| 34 [75]. The colors of things seemed to be changed by sounds or noises. |

Distressing Effects (DisE)

| **Factor 10: Impaired Control and Cognition** |
| --- |
| 1 [8]. I felt like a puppet or marionette. |
| 10 [27]. I was unable to make even the smallest decision. |
| 16 [38]. I had difficulties in distinguishing important from unimportant things. |
| 23 [47]. I felt as if I were paralyzed. |
| 30 [64]. I felt isolated from everything and everyone. |
| 31 [67]. My thoughts kept breaking off; I could not think anything through to the end. |
| 36 [78]. I had the feeling that I no longer had a will of my own. |

| **Factor 11: Anxiety** |
| --- |
| 13 [32]. I was afraid I wouldn’t be able to get out of the state I was in. |
| 20 [43]. I was afraid without being able to say exactly why. |
| 21 [44]. I experienced everything as frighteningly distorted. |
| 22 [46]. I experienced my surroundings as strange and unsettling. |
| 27 [56]. felt threatened. |
| 40 [89]. I had the feeling that something terrible was going to happen. |
