# Supplementary material for: The 3D-ASCr scale: A revalidation of the core dimensions of the Altered States of Consciousness Rating Scale 5D(11)-ASC for psychedelic research
Source: J Psychopharmacol. 2025 Dec 26;40(5):850–62. doi: 10.1177/02698811251397328 (PMC13310268; doi:10.1177/02698811251397328)
Supplement: sj-docx-5-jop-10.1177_02698811251397328 – Supplemental material for The 3D-ASCr scale: A revalidation of the core dimensions of the Altered States of Consciousness Rating Scale 5D(11)-ASC for psychedelic research [file sj-docx-5-jop-10.1177_02698811251397328.docx]

Supplement 5

**Additional Tables and Figure (Dose-level Subscale Means, Eigenvalues, Scree Plot)**

Table S2. Dimension/Subscale means for low, moderate and high dose levels

|  | Aggregated dose levels | | |
| --- | --- | --- | --- |
| Dimension/Subscales | Low | Moderate | High |
| *3D-ASC* | *M (SD)* | *M (SD)* | *M (SD)* |
| OB | 21.6 (21.7) | 37.2 (24.1) | 45.2 (24.4) |
| AED | 7.8 (11.1) | 15.0 (15.4) | 23.5 (20.3) |
| VR | 26.1 (21.9) | 42.5 (23.5) | 48.3 (21.1) |
| *3D-ASCr* |  |  |  |
| Positive Effects | 19.0 (19.5) | 32.2 (22.4) | 39.7 (23.6) |
| Perceptual Effects | 33.8 (29.2) | 53.0 (29.3) | 59.8 (26.2) |
| Distressing Effects | 7.9 (11.2) | 15.7 (16.2) | 23.9 (21.1) |
| *11-ASC* |  |  |  |
| Experience of unity | 19.3 (24.8) | 36.2 (30.2) | 46.3 (31.8) |
| Spiritual experience | 13.9 (21.9) | 21.6 (24.5) | 30.4 (28.3) |
| Blissful state | 30.1 (28.7) | 43.2 (32.2) | 48.4 (34.2) |
| Insightfulness | 15.9 (20.6) | 31.4 (28.6) | 34.1 (29.8) |
| Disembodiment | 18.6 (25.5) | 32.0 (30.8) | 46.0 (34.3) |
| Impaired control and cognition | 12.0 (15.7) | 24.0 (21.5) | 32.7 (24.4) |
| Anxiety | 3.7 (10.2) | 7.4 (15.6) | 15.2 (23.0) |
| Complex imagery | 29.5 (31.0) | 47.1 (33.1) | 53.4 (30.7) |
| Elementary imagery | 40.1 (34.6) | 57.8 (33.5) | 67.0 (29.4) |
| Audiovisual Synesthesia | 31.7 (34.0) | 54.1 (36.4) | 59.0 (35.5) |
| Changed meaning | 15.8 (19.4) | 28.7 (27.1) | 33.2 (27.3) |

Note. To facilitate concise comparison, the sixfold dose-strength classification of Table S1 was condensed into three categories: *low* (low), *moderate* (low-to-moderate, moderate, moderate-to-high), and *high* (high, very high).

Table S3

Initial Eigenvalues of the 11 Factors in the explorative FA

| Factor | Eigenvalue |
| --- | --- |
| 1 | 4.89 |
| 2 | 0.96 |
| 3 | 0.39 |
| 4 | 0.15 |
| 5 | -0.00 |
| 6 | -0.10 |
| 7 | -0.13 |
| 8 | -0.19 |
| 9 | -0.23 |
| 10 | -0.28 |
| 11 | -0.58 |

Figure S1

Scree plot of the explorative FA
